# Multilayered Mechanism of CD4 Downregulation by HIV-1 Vpu Involving Distinct ER Retention and ERAD Targeting Steps

**DOI:** 10.1371/journal.ppat.1000869

**Published:** 2010-04-29

**Authors:** Javier G. Magadán, F. Javier Pérez-Victoria, Rachid Sougrat, Yihong Ye, Klaus Strebel, Juan S. Bonifacino

**Affiliations:** 1 Cell Biology and Metabolism Program, Eunice Kennedy Shriver National Institute of Child Health and Human Development, National Institutes of Health, Bethesda, Maryland, United States of America; 2 Laboratory of Molecular Biology, National Institute of Diabetes and Digestive and Kidney Diseases, National Institutes of Health, Bethesda, Maryland, United States of America; 3 Laboratory of Molecular Microbiology, National Institute of Allergy and Infectious Diseases, National Institutes of Health, Bethesda, Maryland, United States of America; Fred Hutchinson Cancer Research Center, United States of America

## Abstract

A key function of the Vpu protein of HIV-1 is the targeting of newly-synthesized CD4 for proteasomal degradation. This function has been proposed to occur by a mechanism that is fundamentally distinct from the cellular ER-associated degradation (ERAD) pathway. However, using a combination of genetic, biochemical and morphological methodologies, we find that CD4 degradation induced by Vpu is dependent on a key component of the ERAD machinery, the VCP-UFD1L-NPL4 complex, as well as on SCF^β-TrCP^-dependent ubiquitination of the CD4 cytosolic tail on lysine and serine/threonine residues. When degradation of CD4 is blocked by either inactivation of the VCP-UFD1L-NPL4 complex or prevention of CD4 ubiquitination, Vpu still retains the bulk of CD4 in the ER mainly through transmembrane domain interactions. Addition of a strong ER export signal from the VSV-G protein overrides this retention. Thus, Vpu exerts two distinct activities in the process of downregulating CD4: ER retention followed by targeting to late stages of ERAD. The multiple levels at which Vpu engages these cellular quality control mechanisms underscore the importance of ensuring profound suppression of CD4 to the life cycle of HIV-1.

## Author Summary

HIV-1 devotes two accessory proteins, Nef and Vpu, to the task of removing the viral receptor, CD4, from the cell surface. Whereas Nef delivers surface CD4 for degradation in lysosomes, Vpu targets newly-made CD4 in the endoplasmic reticulum for degradation by cytosolic proteasomes. This latter process was thought to be fundamentally distinct from that used for the disposal of abnormal cellular proteins from the endoplasmic reticulum. Contrary to this notion, however, we show that Vpu utilizes at least part of the endoplasmic reticulum-associated degradation machinery to dispose of CD4. Disabling this machinery prevents CD4 degradation induced by Vpu but, surprisingly, does not allow transport of CD4 to the cell surface. This is due to a second function of Vpu: retention of CD4 in the endoplasmic reticulum. These two functions of Vpu are mediated by different parts of the Vpu molecule and involve distinct mechanisms. This functional redundancy underscores the importance of suppressing CD4 expression for HIV-1 to thrive in the infected cells.

## Introduction

Human Immunodeficiency Virus-1 and -2 (HIV-1 and -2), as well as Simian Immunodeficiency Virus (SIV), selectively target helper T-lymphocytes and macrophages/monocytes by binding of their viral envelope protein, Env, to a combination of two cell-type-specific surface receptors: a type 1 transmembrane protein, CD4, and a seven-transmembrane chemokine receptor, CXCR4 or CCR5 [1]. An early and lasting effect of infection is the downregulation of CD4 from the host cell surface [2], [3]. Although it may seem counterproductive for a virus to downregulate its own co-receptor, this event actually promotes the establishment of a robust infection. Indeed, CD4 downregulation prevents (i) superinfection by additional virions [4], (ii) retention of newly-synthesized Env precursor in the endoplasmic reticulum (ER) [5], and (iii) interference with the release of progeny virions from the cell surface [6]. In addition, CD4 downregulation compromises the ability of T-lymphocytes to become activated in response to immunogenic peptides bound to MHC class II molecules on the surface of antigen-presenting cells [7]. These effects all contribute to propagation of the infection, eventually leading to depletion of CD4-positive cells and development of acquired immunodeficiency syndrome (AIDS) in untreated individuals.

The most pathogenic of these viruses, HIV-1, devotes two accessory proteins encoded in its genome, Nef and Vpu, to the task of suppressing CD4 expression [8], [9], [10]. Nef is an N-terminally myristoylated, cytosolically-disposed peripheral membrane protein encoded in the genomes of most strains of HIV-1, HIV-2 and SIV. It is expressed early during infection and functions to accelerate endocytosis of cell surface CD4 by a clathrin/AP-2 pathway [11], [12], [13], followed by delivery of the internalized CD4 to the multivesicular body pathway for eventual degradation in lysosomes [14]. Vpu, on the other hand, is a type III integral membrane protein having a short luminal N-terminal domain (3–12 amino acids), a single transmembrane span that doubles as an uncleaved signal peptide (23 amino acids), and a cytosolic C-terminal domain (47–59 amino acids). Unlike Nef, Vpu is encoded in the genomes of only HIV-1 and a few SIV strains [15]. Vpu is expressed at later stages of infection and acts by targeting newly-synthesized CD4 in the ER for degradation by cytosolic proteasomes [16], [17]. Together, Nef and Vpu ensure profound and sustained suppression of CD4 expression throughout the HIV-1 infectious cycle [18], [19].

CD4 downregulation by Vpu depends on an interaction between the cytosolic domains of both proteins [20]. A canonical DpSGxxpS sequence containing two phosphorylated serine (pS) residues in the cytosolic domain of Vpu (residues number 52 and 56 in the NL4-3 variant of HIV-1 used in this study) then binds β-TrCP1 [21] and β-TrCP2 (also known as FBXW11/HOS) [22], two paralogous F-box adaptor proteins for the cytosolic SCF^β-TrCP^ E3 ubiquitin (Ub) ligase complex. Recruitment of this SCF complex results in ubiquitination of the CD4 cytosolic tail on lysine residues [23], [24], marking CD4 for degradation by cytosolic proteasomes [17]. Unlike CD4, Vpu itself is not ubiquitinated and degraded in this process [25]. Vpu function, therefore, can be likened to that of Ub ligase adaptors, which link substrates to Ub ligases [26].

At first blush, the process of Vpu-induced CD4 degradation evokes the well-known ER-associated degradation (ERAD) pathway, which generally functions to dispose of abnormal proteins from the ER [27], [28], [29]. Two sets of observations, however, distinguish Vpu function from targeting to typical ERAD. First, the cytosolic SCFβ-TrCP complex does not normally function in ERAD, but is responsible for the ubiquitination and degradation of non-ERAD substrates such as IκBα and β-catenin [30], [31]. Instead, the ERAD pathway employs several membrane-bound Ub ligases, including the HRD1-SEL1L complex [32], [33], TEB4/MARCH-VI [34], and the GP78-RMA1 complex [35], [36] (names and references correspond to the mammalian orthologs). Second, genetic analysis involving expression of human CD4 and HIV-1 Vpu in the yeast *S. cerevisiae*, showed that CD4 degradation in the presence of Vpu is independent of components of the ERAD machinery such as Hrd1p, Hrd3p and Ubc7p (yeast names; orthologous to the mammalian proteins HRD1, SEL1L and UBC7, respectively) [23]. These observations have led to the notion that the mechanism by which Vpu induces CD4 degradation is fundamentally distinct from ERAD [23].

We have put this notion to the test by examining the requirement of additional components of the ERAD machinery for Vpu-induced CD4 degradation in human cells. Using siRNA and dominant-negative overexpression approaches, we find a requirement for the VCP-UFD1L-NPL4 complex, which is a key component of the ERAD machinery [37], [38], [39], [40]. In addition, we show that degradation depends on ubiquitination of CD4 on not only lysine, but also serine/threonine residues, the first instance in which a cellular Ub ligase activity is implicated in serine/threonine ubiquitination of an ERAD substrate. Inactivation of the VCP-UFD1L-NPL4 complex prevents degradation of CD4 induced by Vpu. Under these conditions, CD4 accumulates in the ER as a properly folded and membrane-associated protein, indicating that the VCP-UFD1L-NPL4 complex plays a role in the dislocation of CD4 from the ER membrane. Dissection of the mechanism by which Vpu retains CD4 in the ER reveals at least two contributing factors: SCF^β-TrCP^-dependent ubiquitination of the CD4 cytosolic tail and transmembrane domain (TMD) interactions. These findings indicate that Vpu exerts two distinct, separable activities in the process of downregulating CD4: retention in the ER followed by targeting to a variant ERAD pathway.

## Results

### Requirement of the VCP-UFD1L-NPL4 Complex for Vpu-induced CD4 Degradation

**Figure 1:**
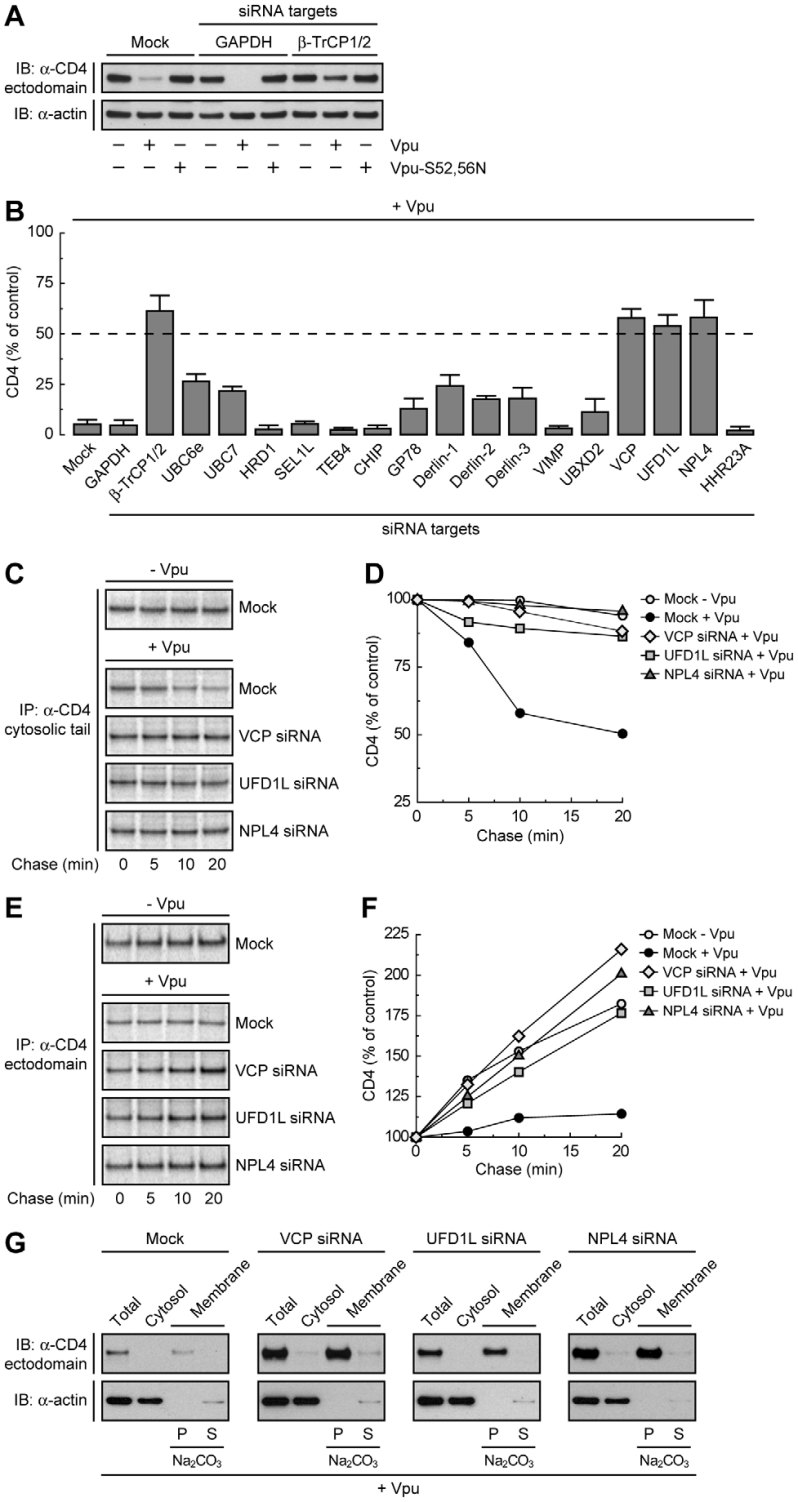
The VCP-UFD1L-NPL4 complex mediates Vpu-dependent CD4 degradation. (A) HeLa cells were treated without (mock) or with siRNAs to GAPDH (negative control) or β-TrCP1/2 (positive control). Cells were then transfected with plasmids encoding human CD4 and no Vpu (empty vector), wild-type Vpu or Vpu-S52,56N. At 12 h after transfection, cell lysates were analyzed by SDS-PAGE and immunoblotting with antibodies to the CD4 ectodomain and actin (used as a loading control). (B) HeLa cells treated with different siRNAs were processed as in A. CD4 levels in the presence of Vpu were quantified by densitometry and expressed as percentage of the total amount of CD4 in the absence of Vpu (100% control). Values represent the mean ± SEM from three independent screens. (C, E) HeLa cells were treated as in A with siRNAs to VCP, UFD1L or NPL4, and then transfected with plasmids encoding human CD4, plus or minus Vpu. At 12 h after transfection, cells were labeled with [^35^S]methionine-cysteine for 2 min and chased for the indicated times at 37°C. Cell extracts were subjected to immunoprecipitation of CD4 using a conformation-independent (C) or conformation-dependent (E) antibody. Immunoprecipitated proteins were resolved by SDS-PAGE and fluorography. (D, F) Percentage of CD4 at each chase time relative to CD4 at time 0 (100% control). (G) A postnuclear supernatant of HeLa cells treated as in C and E was separated into cytosolic and membrane fractions. The membranes were washed with 0.2 M Na_2_CO_3_ pH 11.3, and pellet (P) and supernatant (S) fractions were collected. Fractions were analyzed by SDS-PAGE and immunoblotting with antibodies to the CD4 ectodomain and actin (used as a cytosolic marker).

To determine whether Vpu-induced degradation of CD4 involves any part of the ERAD pathway, we tested for the requirement of key components of the ERAD machinery using a siRNA approach in human cells. To this end, HeLa cells were treated with siRNAs directed to eighteen proteins that mediate various ERAD steps (Table S1) [28]. Human CD4 was then co-expressed with codon-optimized, wild-type HIV-1 Vpu [41] or inactive HIV-1 Vpu bearing mutations of serines 52 and 56 (Vpu-S52,56N) [25] by transient transfection. Total levels of CD4 were determined by immunoblot analysis. A typical immunoblot for negative and positive siRNA controls is shown in Fig. 1A, and quantification of the results from three independent experiments for all the siRNAs tested is shown in Fig. 1B. In cells treated without (mock) or with siRNAs to an irrelevant protein, GAPDH, expression of wild-type Vpu lowered total CD4 levels to <5% of those in control, non-Vpu- or Vpu-S52,56N-expressing cells (Fig. 1, A and B). Combined treatment with siRNAs to β-TrCP1 and β-TrCP2 (β-TrCP1/2) largely protected CD4 from Vpu-induced loss (to ∼60% of control cells), in agreement with previous findings [21], [22]. The remaining siRNA treatments had various effects, with siRNAs to VCP (also known as p97), UFD1L or NPL4 causing the greatest degree of protection (∼60% of control cells). These three cytosolic proteins form a complex that participates in ERAD by extracting or “dislocating” ubiquitinated substrates from the ER membrane [37], [38], [39], [40]. These observations thus pointed to the involvement of at least part of the ERAD pathway in Vpu-induced CD4 degradation.

### Depletion of the VCP-UFD1L-NPL4 Complex Prevents Vpu-induced CD4 Degradation and Results in Accumulation of Folded, Membrane-bound CD4

To ascertain that the prevention of CD4 loss by depletion of VCP, UFD1L or NPL4 was due to a block in CD4 degradation, we performed pulse-chase analysis (Fig. 1, C–F). Cells treated with the corresponding siRNAs and transfected with plasmids encoding CD4, plus or minus Vpu, were labeled for 2 min with [^35^S]methionine-cysteine and chased for different times in complete medium. Immunoprecipitation with a conformation-independent antibody showed that expression of Vpu shortened the half-life of CD4 from ∼4.8 h (Fig. S1, A and B) [16] to ∼20 min in mock-treated cells (Fig. 1, C and D; Fig. S1, C-F). Depletion of VCP, UFD1L or NPL4 largely abrogated the rapid degradation of CD4 induced by Vpu (Fig. 1, C and D; Fig. S1, E and F). Use of a conformation-dependent antibody revealed progressive acquisition of a conformational epitope upon folding of the CD4 ectodomain in the absence of Vpu (Fig. 1, E and F). In mock-treated cells, expression of Vpu prevented accumulation of the folded CD4 species by counteracting folding with degradation (Fig. 1, E and F). Inhibition of degradation by depletion of VCP, UFD1L or NPL4 restored CD4 ectodomain folding (Fig. 1, E and F). Subcellular fractionation and Na_2_CO_3_ treatment showed that the population of CD4 that was protected from Vpu-induced degradation by VCP, UFD1L or NPL4 depletion remained integrally associated with membranes (Fig. 1G). These experiments thus demonstrated that depletion of the VCP-UFD1L-NPL4 complex prevents degradation of CD4 in the presence of Vpu. Under these conditions, the CD4 ectodomain continues to fold while retaining its association with membranes. Moreover, these findings support the notion that the VCP-UFD1L-NPL4 complex is required for extraction of CD4 from membranes.

### The ATPase Activity of VCP is Required for Vpu-induced CD4 Degradation

**Figure 2:**
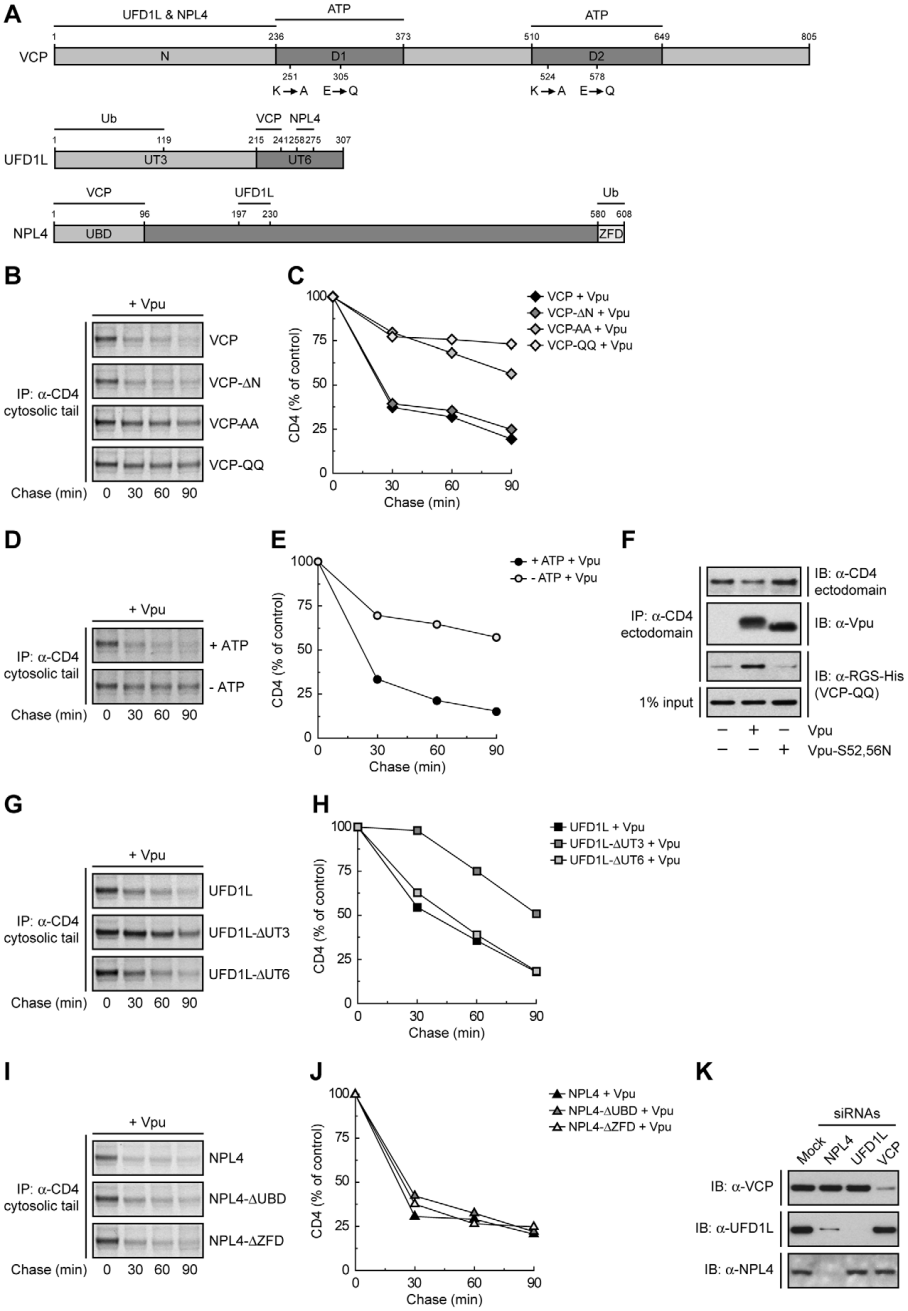
Specific functions for each member of the VCP-UFD1L-NPL4 complex in Vpu-mediated CD4 degradation. (A) Domain structure of mouse VCP, mouse UFD1L and rat NPL4 used in this study. (B, G, I) HeLa cells were transfected with plasmids encoding human CD4, Vpu and wild-type VCP or mutant VCP constructs (VCP-ΔN, VCP-AA, VCP-QQ) (B), wild-type UFD1L or mutant UFD1L constructs (UFD1L-ΔUT3, UFD1L-ΔUT6) (G), or wild-type NPL4 or mutant NPL4 constructs (NPL4-ΔUBD, NPL4-ΔZFD) (I). At 12 h after transfection, cells were labeled with [^35^S]methionine-cysteine for 2 min and chased for the indicated times at 37°C. (D) HeLa cells expressing human CD4 and Vpu were pulse-labeled as described above and chased in normal (+ATP) or ATP-depleted (-ATP) medium. (B, D, G, I) Cell extracts were subjected to immunoprecipitation using an antibody to the CD4 cytosolic tail. Immunoprecipitated proteins were analyzed by SDS-PAGE and fluorography. (C, E, H, J) Percentage of CD4 at each chase time relative to CD4 at time 0 (100% control). (F) HeLa cells were transfected with plasmids encoding human CD4 and RGS-His-tagged VCP-QQ, plus no Vpu (empty vector), wild-type Vpu or Vpu-S52,56N. At 12 h after transfection, cells were lysed and subjected to immunoprecipitation with an antibody to the CD4 ectodomain. Co-precipitation of RGS-His-tagged VCP-QQ with CD4 in the presence of wild-type Vpu was detected by immunoblotting with an antibody to the RGS-His epitope. (K) HeLa cells were treated without (mock) or with siRNAs to VCP, UFD1L or NPL4. Cell extracts were prepared and subjected to immunoblotting with antibodies to VCP, UFD1L or NPL4.

VCP is a member of the AAA-ATPase superfamily; it comprises an N-terminal domain (N) that binds UFD1L and NPL4, and two AAA-ATPase domains (D1 and D2) (Fig. 2A). To investigate the requirement of these domains in Vpu-induced CD4 degradation, we introduced mutations in the VCP cDNA and overexpressed these mutants together with CD4 and Vpu by transfection into HeLa cells. The fate of CD4 was then examined by pulse-chase analysis. VCP-ΔN is a VCP deletion mutant lacking most of the N domain (residues 1–185) (Fig. 2A) [39]. We found that overexpression of this construct had no effect on CD4 degradation (Fig. 2, B and C), probably because this mutant is unable to assemble with the ubiquitin-binding UFD1L and NPL4 proteins, cannot be targeted to ubiquitinated substrates, and does not compete with endogenous VCP. Mutation of lysine and glutamate residues in the active sites of both ATPase domains to alanine (VCP-AA) or glutamine (VCP-QQ) residues, respectively (Fig. 2A), is known to prevent ATP binding (VCP-AA) or ATP hydrolysis (VCP-QQ) [39]. Because of the presence of the N domain, these constructs are capable of assembling with UFD1L and NPL4, allowing the recruitment of catalytically-inactive VCP to ubiquitinated substrates. The VCP-AA mutant was previously shown to abrogate Vpu-induced CD4 degradation [24]. We confirmed this observation and, in addition, found that the VCP-QQ had a similar effect (Fig. 2, B and C), indicating that these mutants exerted a potent dominant-negative effect, and that both ATP binding and hydrolysis are required for this process. VCP is thus likely to provide the energy required for extraction of CD4 from membranes. In line with this conclusion, depletion of ATP by incubation of cells in glucose-free medium supplemented with 2-deoxy-D-glucose and sodium azide [42] inhibited CD4 degradation induced by Vpu (Fig. 2, D and E). Co-precipitation experiments showed that the substrate-trapping VCP-QQ mutant was isolated as a complex with CD4 in the presence of wild-type Vpu but not Vpu-S52,56N (Fig. 2F). Because these Vpu constructs differ in their ability to promote CD4 ubiquitination by the SCF^β-TrCP^ complex (Fig. S2), our findings are consistent with recruitment of VCP to ubiquitinated CD4.

### Ub Binding to UFD1L, but not NPL4, is Important for CD4 Degradation by Vpu

The recruitment of VCP to CD4 is likely mediated by UFD1L and NPL4. These proteins form a heterodimer that assembles with a VCP homohexamer [43]. Both UFD1L and NPL4 contain binding sites for each other and for VCP (Fig. 2A) [44], [45]. In addition, UFD1L and NPL4 contain domains that bind K48-linked and K63-linked Ub chains, respectively (Fig. 2A) [39], [46]. Overexpression of an UFD1L mutant lacking the binding site for VCP and NPL4 (UFD1L-ΔUT6) had no effect on Vpu-mediated CD4 degradation (Fig. 2, G and H), probably because it cannot interfere with the ability of endogenous UFD1L to engage VCP and NPL4 for interaction with ubiquitinated CD4. Overexpression of an UFD1L mutant lacking the Ub-binding domain (UFD1L-ΔUT3), on the other hand, inhibited Vpu-induced CD4 degradation (Fig. 2, G and H), whereas overexpression of an analogous NPL4 mutant (NPL4-ΔZFD) had no effect on this process (Fig. 2, I and J). The dominant-negative effect of UFD1L-ΔUT3 might be explained by the ability of this mutant to bind both VCP and NPL4 but not be recruited to proteins conjugated to K48-linked Ub chains. This is consistent with the specific involvement of K48 Ub linkages in ERAD [39]. We also observed that overexpression of a NPL4 mutant lacking the VCP-binding domain (NPL4-ΔUBD) failed to elicit a dominant-negative effect (Fig. 2, I and J). The inability of these NPL4 mutants to exert a dominant-negative effect stands in sharp contrast with the inhibitory effect of the NPL4 siRNAs on Vpu-induced CD4 degradation (Fig. 1, B-D). The explanation for this apparent discrepancy lay on the effects of each siRNA on the levels of the three components of the complex (Fig. 2K). Indeed, siRNAs to VCP, UFD1L or NPL4 resulted in depletion of the corresponding target protein without affecting the levels of the other two, with the notable exception of the NPL4 siRNAs, which depleted both NPL4 and UFD1L (Fig. 2K). This indicated that NPL4 is required to stabilize UFD1L, as previously reported [47]. Taken together, these findings ascribe specific functions to each of the components of the VCP-UFD1L-NPL4 complex in Vpu-mediated CD4 degradation: VCP energizes the process through ATP binding and hydrolysis, UFD1L binds ubiquitinated CD4 through recognition of K48 Ub chains, and NPL4 stabilizes UFD1L.

### Vpu Also Mediates Retention of CD4 in the ER

**Figure 3:**
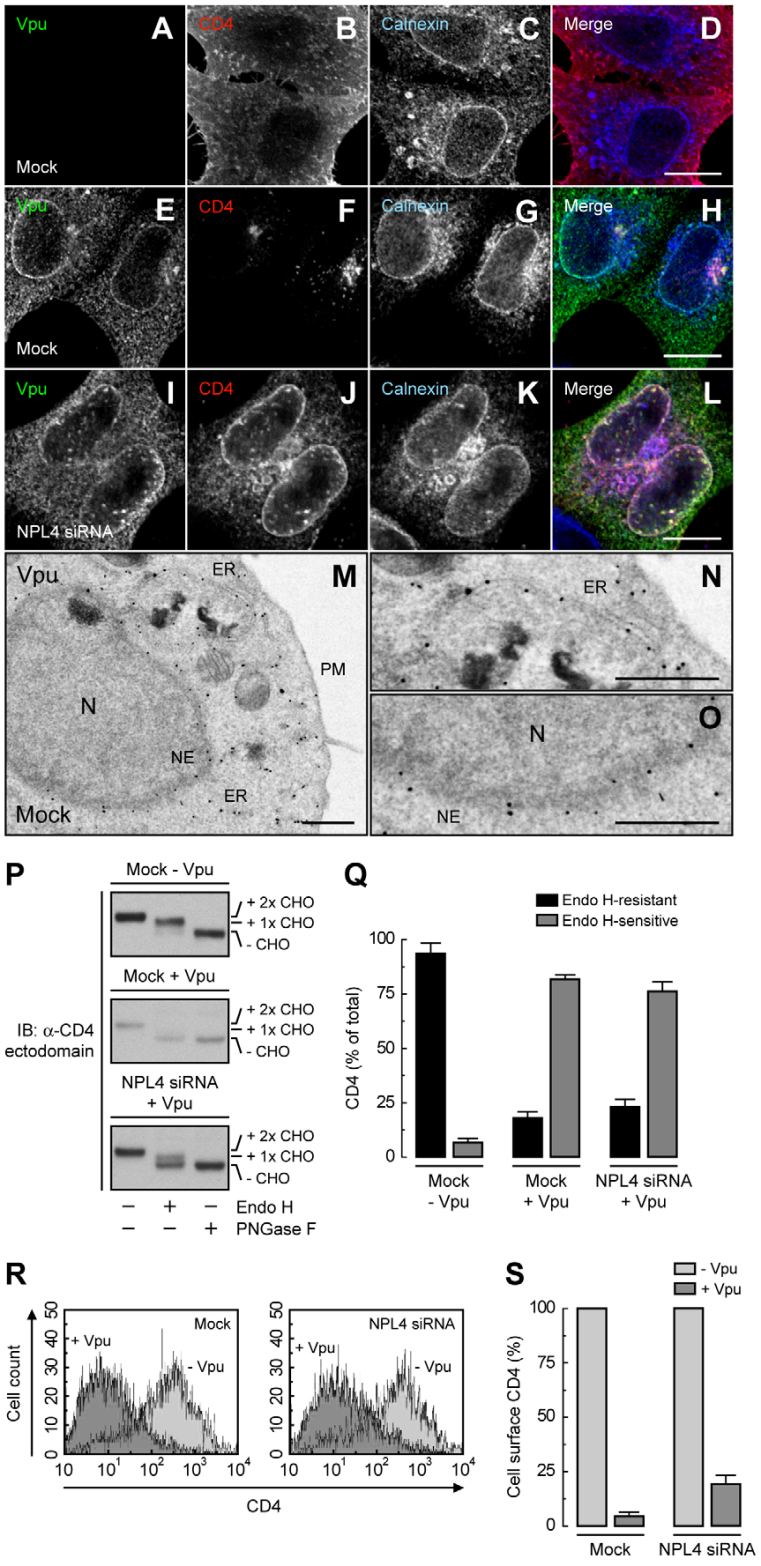
ER retention of CD4 mediated by Vpu. (A–L) HeLa cells were treated without (mock) (A–H) or with siRNAs to NPL4 (I–L). Cells were then transfected with plasmids encoding human CD4 without (A–D) or with Vpu (E–L). At 12 h after transfection, cells were fixed, permeabilized and labeled with a rabbit polyclonal antibody to Vpu (green channel; A, E, I), IgG_2a_ mouse monoclonal antibody to CD4 (red channel; B, F, J) and IgG_1_ mouse monoclonal antibody to calnexin (blue channel; C, G, K). Stained cells were examined by confocal fluorescence microscopy. Bars: 10 µm. (M–O) HeLa cells expressing Vpu were fixed and processed for immunoelectron microscopy as previously described [14]. Notice that enhanced nanogold particles indicative of Vpu are mainly associated with the ER and nuclear envelope (NE). In contrast, untransfected cells show no enhanced nanogold, confirming the specificity of the staining (Fig. S3). N: nucleus; PM: plasma membrane. Bars: 1 µm. (P) Total lysates from HeLa cells treated as in A–L were digested with Endo H, PNGase F or left untreated before immunoblotting with an antibody to the CD4 ectodomain. CHO: N-linked carbohydrate chain. (Q) Data are represented as mean ± SEM from three independent experiments like that in P. (R) HeLa cells treated as in A–L were analyzed for cell surface CD4 by FACS. (S) Bar graphs represent percentage of surface CD4 levels in cells expressing Vpu relative to CD4-surface levels in the absence of Vpu (100%). Values are expressed as mean ± SEM from three independent experiments.

We next wondered whether inhibition of Vpu-induced CD4 degradation by interference with the VCP-UFD1L-NPL4 complex allowed transport of newly-synthesized CD4 out of the ER. To assess this possibility, we performed siRNA-mediated depletion of NPL4 (which, as described above, depleted both NPL4 and UFD1L), expressed CD4 in the absence or presence of Vpu, and examined the localization of CD4 by immunofluorescence microscopy (Fig. 3, A-L), sensitivity to Endo H (Fig. 3, P and Q), and FACS analysis (Fig. 3, R and S). Immunofluorescence microscopy showed that CD4 was predominantly at the plasma membrane in the absence of Vpu (Fig. 3B). As expected, expression of Vpu, which localized to the ER as well as juxtanuclear structures corresponding to the *trans*-Golgi network and endosomes (Fig. 3, E and M–O; Fig. S3, A–C), caused a marked loss of CD4 staining in mock-treated cells (Fig. 3F). Interestingly, in NPL4-depleted cells, both Vpu (Fig. 3I) and the protected CD4 (Fig. 3J) co-localized on the ER, as evidenced by co-staining for the ER-resident protein, calnexin (Fig. 3K). Normally, newly-synthesized CD4 receives two N-linked high-mannose oligosaccharide chains, only one of which acquires complex carbohydrates upon transport through the Golgi complex [16]. Treatment with Endo H removes one high-mannose chain, whereas treatment with PNGase F removes both chains, as detected by SDS-PAGE and immunoblot analysis of total CD4 (Fig. 3P, top panel; Fig. 3Q). The small amount of CD4 that remained upon expression of Vpu in mock-treated cells was completely sensitive to Endo H, indicating that it was localized to the ER (Fig. 3P, middle panel; Fig. 3Q). This phenotype was not due to a general impairment of protein maturation through the biosynthetic pathway because acquisition of Endo H-resistance by the transferrin receptor was not affected by expression of Vpu (Fig. S3, D and E). Significantly, in NPL4-depleted, Vpu-expressing cells, ∼75% of CD4 remained sensitive to Endo H (Fig. 3P, lower panel; Fig. 3Q), consistent with localization of the majority of CD4 to the ER. Finally, FACS analysis showed that depletion of NPL4 *per se* did not alter expression of CD4 at the cell surface in the absence of Vpu (Fig. 3, R and S). However, in line with the experiments described above, Vpu drastically reduced (∼80%) surface CD4 expression even in NPL4-depleted cells (Fig. 3, R and S). Altogether, these results demonstrated that a large fraction of CD4 remains in the ER in the presence of Vpu when targeting for degradation is blocked. Therefore, Vpu mediates CD4 retention in the ER in addition to degradation.

### Binding of β-TrCP1/2 to Vpu Contributes to CD4 Retention in the ER

**Figure 4:**
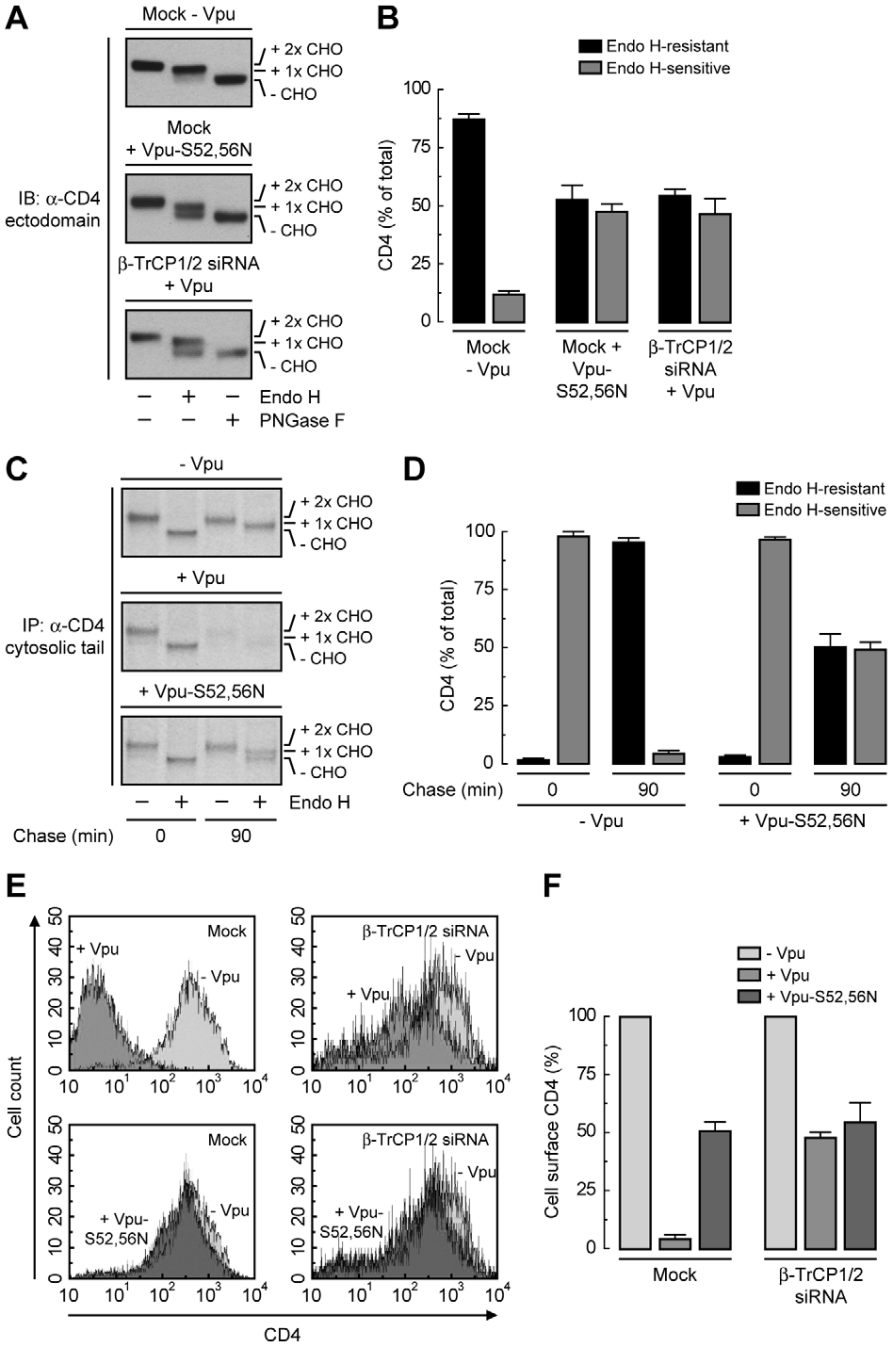
SCF^β-TrCP^ recruitment by Vpu is required for CD4 retention in the ER. (A) HeLa cells were treated without (mock) or with siRNAs to β-TrCP1/2. Cells were then transfected with plasmids encoding human CD4 and no Vpu (empty vector), wild-type Vpu or Vpu-S52,56N. The glycosylation state of CD4 was analyzed as described in the legend to Fig. 3P. (B) Data are represented as mean ± SEM from three independent experiments like that in A. (C) HeLa cells expressing human CD4 in the absence or presence of wild-type Vpu or Vpu-S52,56N were labeled with [^35^S]methionine-cysteine for 2 min and chased for the indicated times at 37°C. Cell extracts were subjected to immunoprecipitation with an antibody to the CD4 cytosolic tail. Immunoprecipitated proteins were digested with Endo H or left untreated before analysis by SDS-PAGE and fluorography. (D) Data from three independent experiments like that in C are represented as mean ± SEM. (E) HeLa cells treated as in A were analyzed for cell surface CD4 by FACS. (F) Bar graphs represent percentage of surface CD4 levels in cells expressing wild-type Vpu or Vpu-S52,56N relative to CD4-surface levels in the absence of Vpu (100%). Values are expressed as mean ± SEM from three independent experiments.

To dissect the mechanism by which Vpu causes CD4 retention in the ER, we examined the effect of disrupting the Vpu-β-TrCP1/2 interaction by either mutating serines 52 and 56 to asparagine in Vpu [21], [25] or depleting cells of both β-TrCP1 and β-TrCP2 [22]. A quantitative measure of ER retention was obtained by Endo H treatment in conjunction with immunoblotting (Fig. 4, A and B) or pulse-chase analysis (Fig. 4, C and D). Using these assays, we found that ∼50% of CD4 was retained in the ER upon disruption of the Vpu-β-TrCP1/2 interaction by either method. Consistent with these observations, FACS analysis showed that expression of Vpu-S52,56N, depletion of β-TrCP1/2, or a combination of both, resulted in ∼50% reduction of CD4 surface levels (Fig. 4, E and F). Comparison to the retention observed upon NPL4 depletion (∼75%) (Fig. 3, P–S) indicated that binding of β-TrCP1/2 to Vpu contributes to CD4 retention in the ER, but is probably not the only factor.

### Lysine and Serine/Threonine Residues in the Cytosolic Tail of CD4 Contribute to Ubiquitination, Degradation and ER Retention

**Figure 5:**
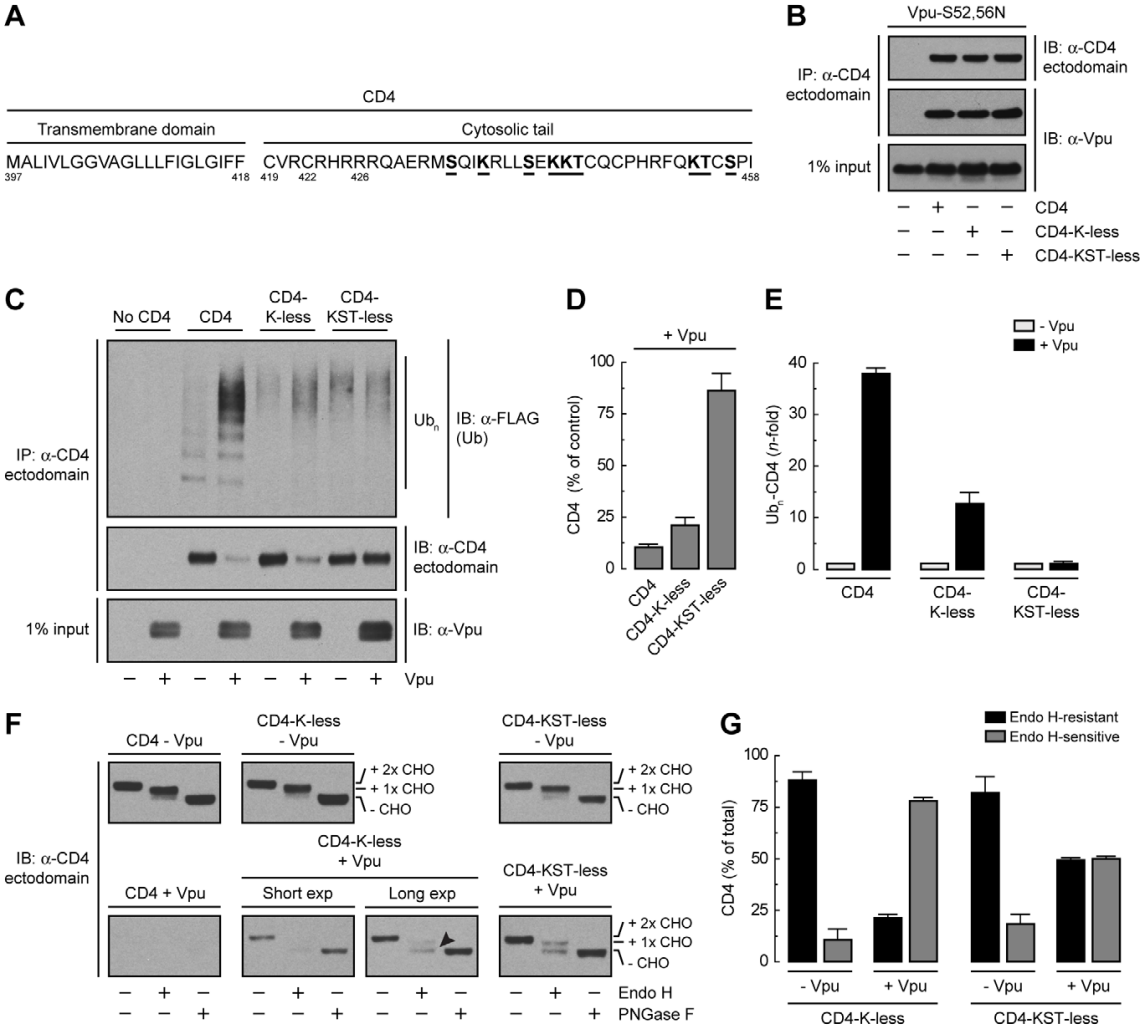
Requirement of lysine and serine/threonine residues in the cytosolic tail of CD4 for ubiquitination, degradation and ER retention. (A) Sequence of the CD4 TMD and cytosolic tail with residues targeted for mutagenesis underlined. (B) HeLa cells were transfected with plasmids encoding Vpu-S52,56N and no CD4 (empty vector), human wild-type CD4, CD4 with all four cytosolic lysine residues mutated to arginine (CD4-K-less), or CD4 with all cytosolic lysine, serine and threonine residues mutated to arginine, alanine and isoleucine, respectively (CD4-KST-less). At 12 h after transfection, cells were lysed and subjected to immunoprecipitation with an antibody to the CD4 ectodomain and immunoblotting with an antibody to Vpu. (C) HeLa cells were transfected with plasmids encoding no CD4 (empty vector), human wild-type CD4, CD4-K-less or CD4-KST-less and FLAG-tagged Ub, plus or minus Vpu (1∶0.5∶1 ratio of CD4, Ub and Vpu, respectively). At 12 h after transfection, equivalent amounts of cell lysates made under denaturing conditions were subjected to immunoprecipitation with a conformation-independent antibody to the CD4 ectodomain. Ubiquitination of CD4 was detected by immunoblotting with a polyclonal antibody to the FLAG epitope. (D) CD4 levels in the presence of Vpu from C were quantified by densitometry and expressed as percentage of the total amount of CD4 in the absence of Vpu (100% control). Data are represented as the mean ± SEM from three independent experiments. (E) Ub_n_-CD4 levels in the presence of Vpu from C were quantified by densitometry and expressed as percentage of the total amount of Ub_n_-CD4 in the absence of Vpu (100% control). These values were normalized for the remaining CD4 in C (*i.e.*, 1 for Ub_n_-CD4 in the absence of Vpu). Data are the mean ± SEM from three independent experiments. (F) Cell lysates from HeLa cells expressing human CD4, CD4-K-less or CD4-KST-less, plus or minus Vpu, were digested with Endo H, PNGase F or left untreated before immunoblotting with an antibody to the CD4 ectodomain. The arrowhead indicates Endo H-sensitive species corresponding to the CD4-K-less mutant in the presence of Vpu. (G) Data from three independent experiments such as that in F are represented as the mean ± SEM.

Binding of β-TrCP1/2 to Vpu could contribute to CD4 retention in the ER directly through assembly of a transport-incompetent complex or indirectly through ubiquitination mediated by the SCF^β-TrCP^ complex. To examine the role of ubiquitination in ER retention and degradation, we mutated all potential Ub-acceptor lysine residues to arginine (CD4-K-less) or lysine, serine and threonine residues to arginine, alanine and isoleucine, respectively (CD4-KST-less), in the cytosolic tail of CD4 (Fig. 5A). None of these mutations affected the ability of CD4 to interact with Vpu-S52,56N, as determined by co-precipitation analysis (Fig. 5B). Ubiquitination was assessed by co-expression of untagged CD4 constructs with FLAG-tagged Ub in the absence or presence of Vpu. Cell extracts were fully denatured prior to immunoprecipitation with a conformation-independent antibody to a luminal CD4 epitope and immunoblotting with an antibody to the FLAG epitope (Fig. 5C). This protocol ensured that ubiquitinated species corresponded to CD4 and not to associated proteins. The amounts of ubiquitinated CD4 were normalized to the amounts of remaining CD4 in each sample (Fig. 5, D and E). We observed that expression of Vpu enhanced ubiquitination of CD4 ∼39-fold (Fig. 5, C and E). Mutation of all four cytosolic lysines decreased but did not completely abolish CD4 ubiquitination (Fig. 5, C and E) and only slightly diminished degradation (Fig. 5, C and D) induced by Vpu. Additional mutation of the cytosolic serine and threonine residues abrogated virtually all ubiquitination (Fig. 5, C and E) and degradation (Fig. 5, C and D) induced by Vpu. These results indicated that targeting of CD4 for degradation depends on ubiquitination of lysine and serine/threonine residues in the cytosolic tail.

Endo H digestion analysis showed that the CD4-K-less and CD4-KST-less mutants were normally exported from the ER in the absence of Vpu (Fig. 5, F and G). Expression of Vpu, however, resulted in retention of ∼80% of the CD4-K-less mutant in the ER (Fig. 5, F and G), similarly to wild-type CD4 (Fig. 3, P and Q). Interestingly, the CD4-KST-less mutant was retained ∼50% in the ER (Fig. 5, F and G). This level of retention is similar to that observed upon disruption of the Vpu-β-TrCP1/2 interaction (Fig. 4, A and B), indicating that the contribution of this interaction to ER retention is likely indirect, through SCF^β-TrCP^–mediated ubiquitination of the CD4 cytosolic tail.

### TMD Interactions Are the Main Determinant of ER Retention of CD4 by Vpu

**Figure 6:**
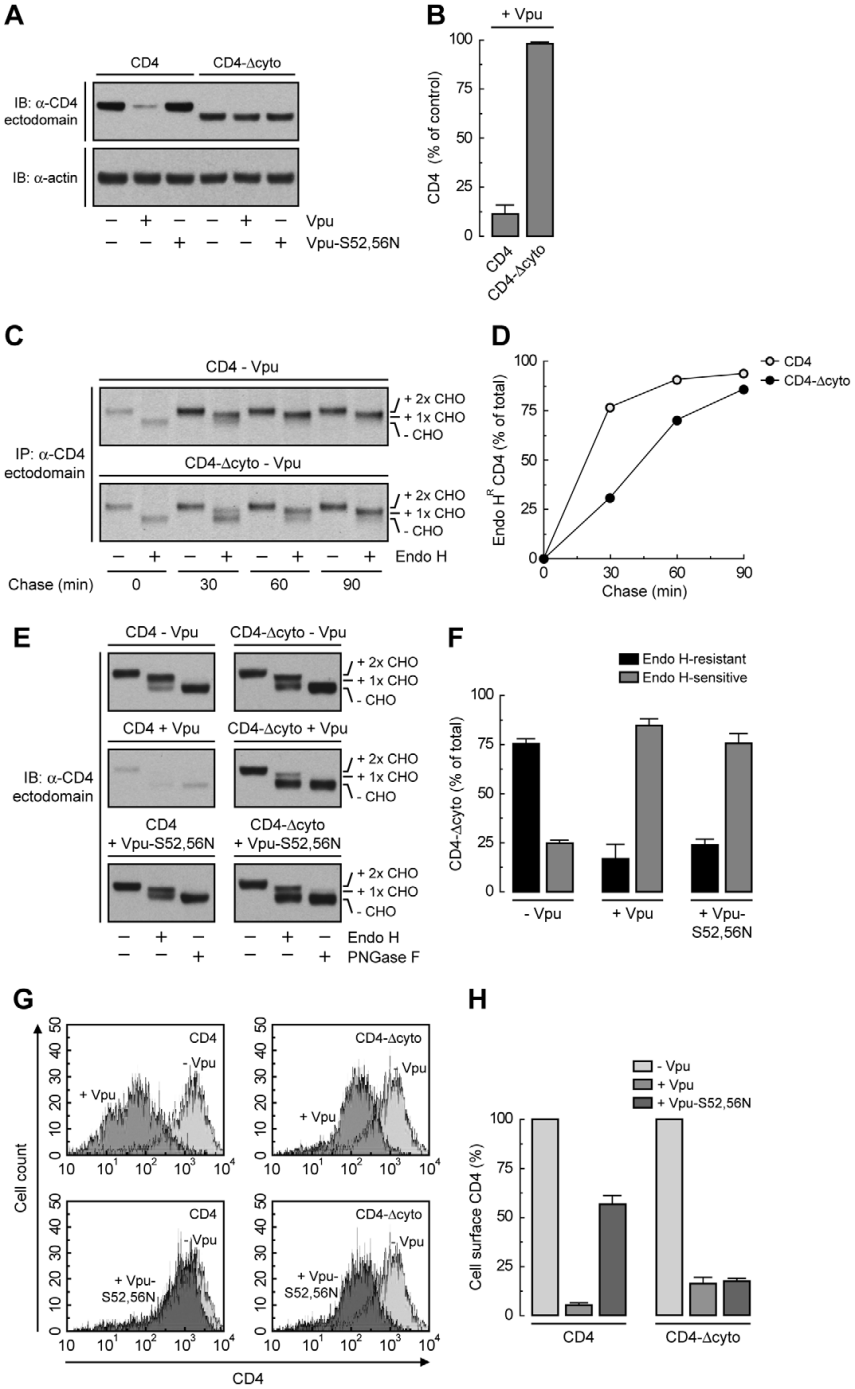
Deletion of CD4 cytosolic tail prevents degradation but not ER retention induced by Vpu. (A) HeLa cells were transfected with plasmids encoding human CD4 or CD4-Δcyto together with no Vpu (empty vector), wild-type Vpu or Vpu-S52,56N. At 12 h after transfection, cell extracts were prepared and subjected to immunoblotting with an antibody to the CD4 ectodomain. (B) CD4 levels in the presence of wild-type Vpu were quantified by densitometry and expressed as percentage of the total amount of CD4 in the absence of Vpu (100% control). Data are represented as the mean ± SEM from three independent experiments. (C) HeLa cells expressing human CD4 or CD4-Δcyto in the absence of Vpu were labeled with [^35^S]methionine-cysteine for 2 min and chased for the indicated times at 37°C. Cell extracts were subjected to immunoprecipitation with an antibody to the CD4 ectodomain. Immunoprecipitated proteins were digested with Endo H or left untreated before analysis by SDS-PAGE and fluorography. The increased detection of both CD4 constructs from 0 to 30 min of chase is due to the development of a conformational epitope recognized by this particular antibody. (D) Data from C are plotted as percentage of Endo H-resistant (Endo H^R^) CD4 as a function of the chase time. (E) Total cell lysates from HeLa cells treated as in A were digested with Endo H, PNGase F or left untreated before immunoblotting with an antibody to the CD4 ectodomain. (F) Data from three independent experiments like that in E are represented as mean ± SEM. (G) HeLa cells treated as in A were analyzed for cell surface CD4 by FACS. (H) Bar graphs represent percentage of surface CD4 levels in cells expressing wild-type Vpu or Vpu-S52,56N relative to CD4-surface levels in the absence of Vpu (100%). Values are expressed as mean ± SEM from three independent experiments.

The fact that Vpu still retains ∼50% of CD4 in the ER independently of the interaction of Vpu with β-TrCP1/2 or the ubiquitination of the CD4 cytosolic tail suggests an additional role for the CD4-Vpu interaction in ER retention of CD4. This interaction has been previously shown to depend on the cytosolic domains of both CD4 and Vpu [20]. To test whether this particular interaction accounted for the bulk of ER retention, we examined the effect of deleting most of the cytosolic tail from CD4 (residues 426–458) (CD4-Δcyto construct; Fig. 5A). This truncation should eliminate interaction with Vpu and, consequently, all retention and degradation determinants. As expected, CD4-Δcyto was not degraded in the presence of Vpu (Fig. 6, A and B) [20]. Pulse-chase experiments combined with Endo H digestion showed that CD4-Δcyto completely exited the ER, albeit more slowly than full-length CD4, in the absence of Vpu (Fig. 6, C and D). These results indicated that the CD4 cytosolic tail has only a weak ER export signal. Surprisingly, expression of Vpu resulted in ∼80% retention of CD4-Δcyto in the ER (Fig. 6, E and F). Similar results were obtained with a CD4 construct having a more radical deletion of the cytosolic domain (residues 422–458) (Fig. 5A) (data not shown). Therefore, interactions other than those between the cytosolic domains must be the main determinants of Vpu-mediated retention of CD4 in the ER. Since Vpu has a very short and variable luminal domain (3–12 non-conserved amino acids; 4 in the HIV-1 NL4-3 variant used in this study), direct or indirect interactions at the level of the TMDs must be the main determinant of the CD4 retention in the ER mediated by Vpu. The fact that ER retention of CD4-Δcyto by Vpu-S52,56N was greater (∼75%) (Fig. 6, E and F) than that of full-length CD4 (∼50%) (Fig. 4, A and B) further supports the occurrence of a weak ER export signal in the CD4 cytosolic tail. These effects at the level of the ER were reflected in changes in surface expression of CD4 analyzed by FACS (Fig. 6, G and H). Thus, Vpu has an intrinsic ability to retain CD4 in the ER that is independent of the cytosolic domain of CD4 and is likely mediated by TMD interactions.

**Figure 7:**
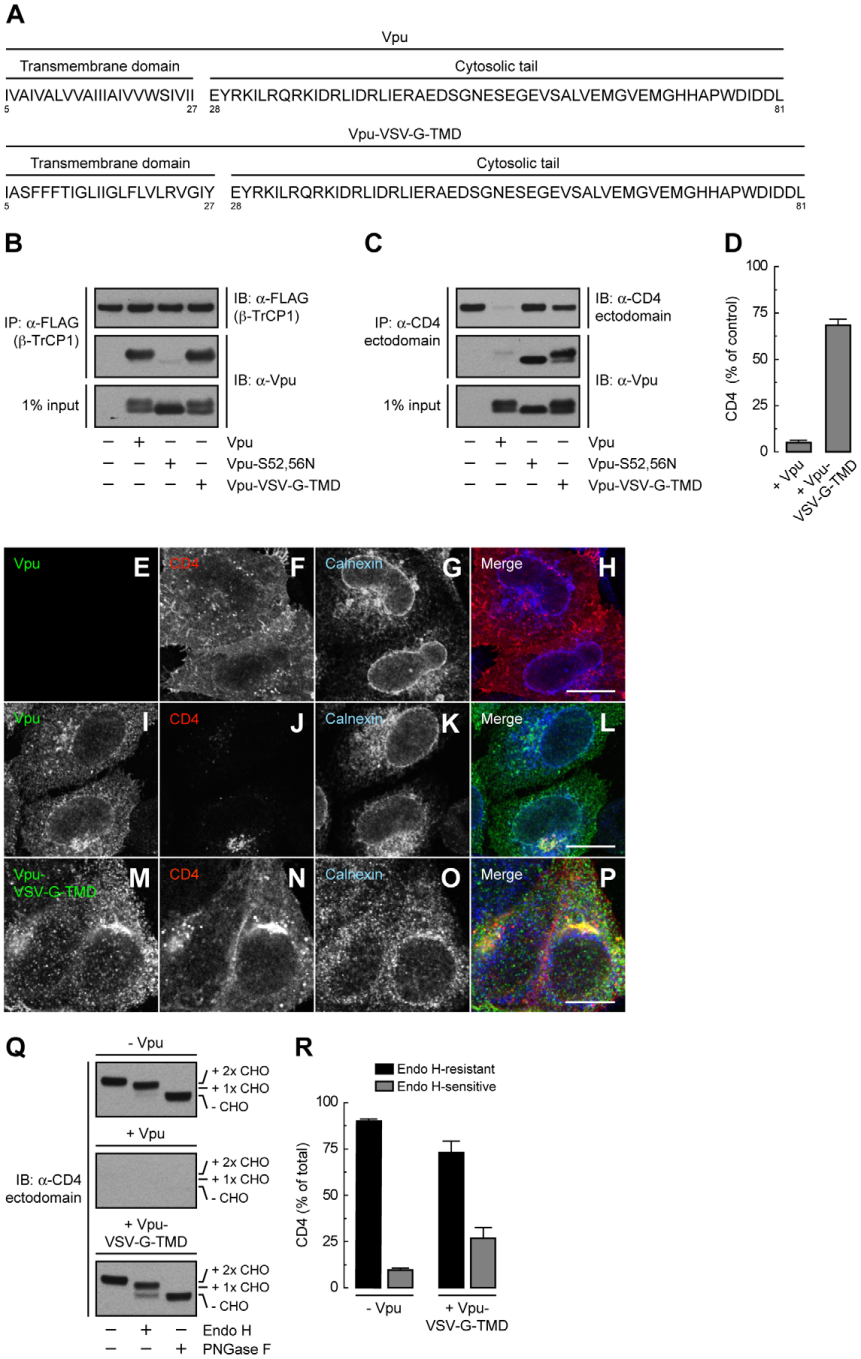
The Vpu TMD is required for induction of both ER retention and degradation of CD4. (A) Amino acid sequence of the TMD and cytosolic tail of wild-type Vpu and Vpu-VSV-G-TMD. (B) HeLa cells were transfected with plasmids encoding FLAG-tagged human β-TrCP1, plus no Vpu (empty vector), wild-type Vpu, Vpu-S52,56N or Vpu-VSV-G-TMD. At 12 h after transfection, cells were lysed and subjected to immunoprecipitation using an antibody to the FLAG epitope. Co-precipitation of the different Vpu variants with β-TrCP1 was detected by immunoblotting with an antibody to Vpu. Notice that only phosphorylatable wild-type Vpu and Vpu-VSV-G-TMD co-precipitate with β-TrCP1. (C) Detergent lysates from HeLa cells expressing human CD4 in the absence or presence of wild-type Vpu, Vpu-S52,56N or Vpu-VSV-G-TMD were immunoprecipitated with an antibody to the CD4 ectodomain. Vpu co-precipitation with CD4 was detected as described in B. (D) CD4 levels in the presence of wild-type Vpu or Vpu-VSV-G-TMD were quantified by densitometry and expressed as percentage of the total amount of CD4 in the absence of Vpu (100% control). Values are the mean ± SEM from three independent experiments. (E–P) HeLa cells were transfected with plasmids encoding human CD4 and no Vpu (empty vector; E–H), wild-type Vpu (I–L) or Vpu-VSV-G-TMD (M–P). At 12 h after transfection, cells were fixed, permeabilized and labeled with a rabbit polyclonal antibody to Vpu (green channel; E, I, M), IgG_2a_ mouse monoclonal antibody to CD4 (red channel; F, J, N) and IgG_1_ mouse monoclonal antibody to calnexin (blue channel; G, K, O). Stained cells were examined by confocal fluorescence microscopy. Bars: 10 µm. (Q) Total lysates from HeLa cells treated as in E–P were digested with Endo H, PNGase F or left untreated before immunoblotting with an antibody to the CD4 ectodomain. (R) Data are represented as mean ± SEM from three independent experiments like that in Q.

To assess more directly the importance of the Vpu TMD, we replaced it with that of the G protein of vesicular stomatitis virus (VSV-G), resulting in a chimera designated Vpu-VSV-G-TMD (Fig. 7A). Co-precipitation experiments showed that Vpu-VSV-G-TMD interacted with β-TrCP1 (Fig. 7B) and CD4 (Fig. 7C) to the same extent as wild-type Vpu. However, this chimera largely failed to promote CD4 degradation (Fig. 7, C and D). This observation is in line with previous reports that substitutions of the Vpu or CD4 TMDs abrogate Vpu-induced CD4 degradation [48], [49]. The TMDs must therefore play a specific role in ERAD, perhaps in allowing dislocation of CD4 from the ER membrane. Immunofluorescence microscopy (Fig. 7, E–P) and Endo H digestion analysis (Fig. 7, Q and R) showed that Vpu-VSV-G-TMD also failed to prevent exit of the bulk of CD4 from the ER towards the plasma membrane. Only a minor fraction of CD4 (∼25%) remained in the ER in the presence of Vpu-VSV-G-TMD (Fig. 7, Q and R), presumably because of cytosolic domain interactions with Vpu/β-TrCP1/2 and ensuing ubiquitination (Figs. 4 and 5). Taken together, these results indicate that TMD interactions play important roles in Vpu-dependent ER retention and degradation of CD4.

### A Strong ER Export Signal Overrides ER Retention of CD4 Mediated by Vpu

**Figure 8:**
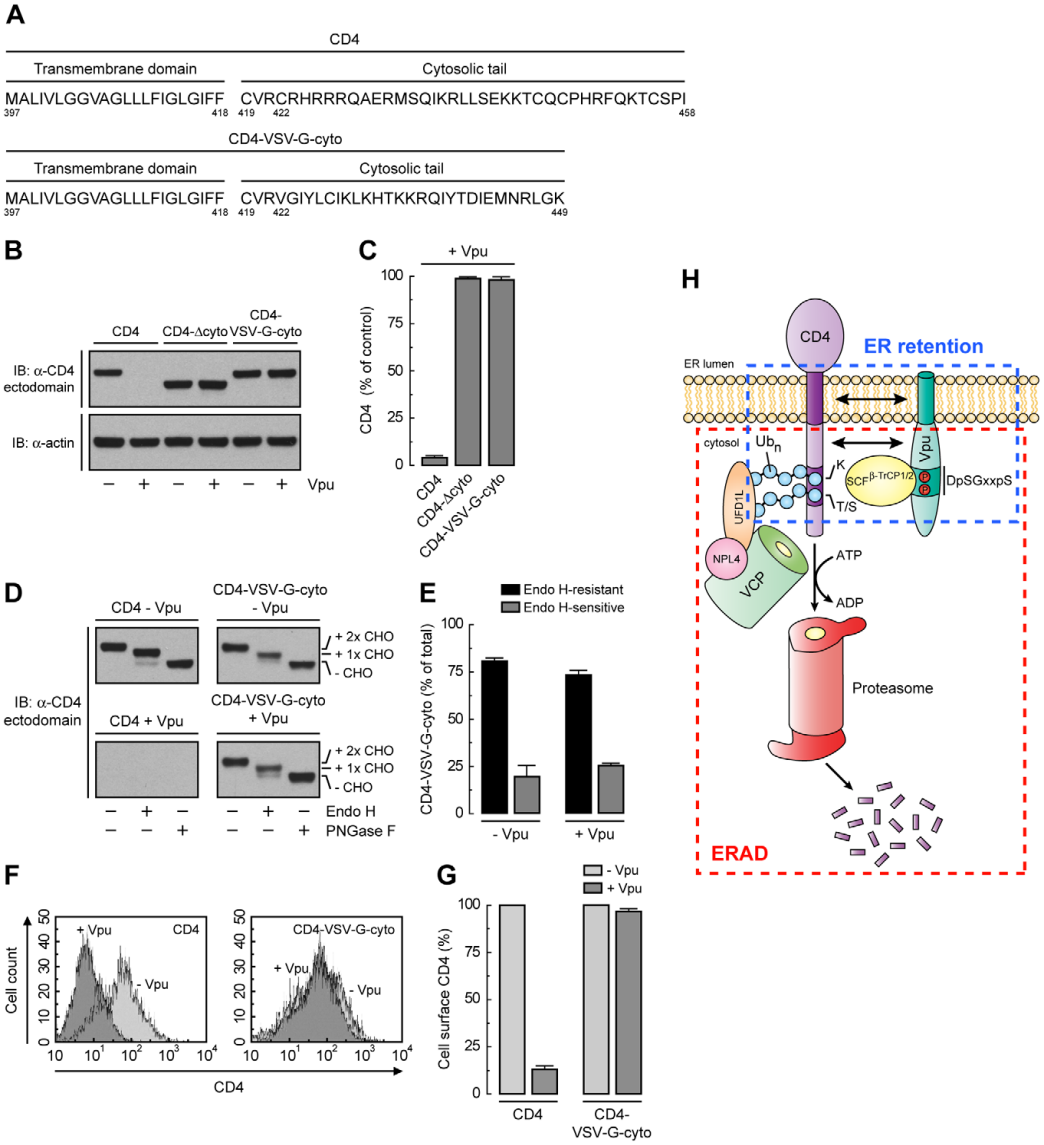
A strong ER export signal overrides ER retention of CD4 by Vpu. (A) Amino acid sequence of the TMD and cytosolic tail of wild-type CD4 and CD4-VSV-G-cyto. (B, C) The effect of Vpu on human CD4, CD4-Δcyto or CD4-VSV-G-cyto was analyzed as described for Fig. 6, A and B. (D, E) The carbohydrate processing of CD4 and CD4-VSV-G-cyto in the presence or absence of Vpu was analyzed as described for Fig. 6, E and F. (F, G) HeLa cells expressing human CD4 or CD4-VSV-G-cyto, plus or minus Vpu, were analyzed for cell surface CD4 by FACS as described for Fig. 6, G and H. (H) Model for the dual role of Vpu in ER retention and ERAD targeting of CD4. See Discussion for details.

Unlike CD4, VSV-G has a strong ER export signal in its cytosolic tail [50]. Substitution of the VSV-G cytosolic tail for the CD4 cytosolic tail resulted in a chimeric protein (CD4-VSV-G-cyto mutant; Fig. 8A) that was not degraded in the presence of Vpu (Fig. 8, B and C), presumably because the VSV-G cytosolic tail does not interact with Vpu and lacks degradation determinants. Endo H digestion showed that, in contrast to CD4-Δcyto, this chimera was efficiently transported out of the ER both in the absence or presence of Vpu (Fig. 8, D and E). In addition, FACS analysis showed that this chimera was expressed at the cell surface very efficiently irrespective of the presence or absence of Vpu (Fig. 8, F and G). Therefore, the VSV-G export signal is capable of overriding ER retention of CD4 mediated by Vpu.

## Discussion

### Dual Functions of Vpu in ER Retention and ERAD Targeting of CD4

The results of our study shed light on two aspects of Vpu function that remained poorly understood: its ability to retain CD4 in the ER and to target CD4 to the ERAD pathway. The scheme shown in Fig. 8H represents our view of how these aspects are integrated. We think that Vpu first acts to retain CD4 in the ER (Fig. 3) by virtue of TMD interactions (Figs. 6 and 7). The cytosolic domain of Vpu then interacts with CD4 and recruits the SCF^β-TrCP^ Ub ligase complex (Fig. 4) [21], [22], which mediates the addition of multiple Ub moieties to lysine [23], [24] and serine/threonine residues (Fig. 5) in the cytosolic tail of CD4. Ubiquitination further contributes to CD4 retention in the ER, and additionally marks CD4 for delivery to proteasomes (Fig. 5). This delivery involves recruitment of the VCP-UFD1L-NPL4 complex through recognition by UFD1L of K48-linked poly-Ub chains on the CD4 cytosolic tail (Figs. 1 and 2). The ATPase activity of VCP then drives dislocation of CD4 from the ER membrane into the cytosol (Fig. 2) [24] for eventual degradation in proteasomes [17]. The multiple levels at which Vpu acts to prevent export of CD4 from the ER underscore the importance of ensuring complete suppression of CD4 for progression of the infection.

### The Mechanism of ER Retention

Our experiments show that a large fraction (∼50-80%) of CD4 remains in the ER in the presence of Vpu when ERAD is blocked by inactivation of the VCP-UFD1L-NPL4 complex (Fig. 3), disruption of the Vpu-β-TrCP1/2 interaction (Fig. 4), or mutation of lysine, serine and threonine residues in the cytosolic tail of CD4 (Fig. 5), in all cases in the absence of Env or any other inhibitor of ER export. Furthermore, this retention is independent of the only interaction between Vpu and CD4 reported to date, which involves the cytosolic domains of both proteins [20]. Indeed, deletion of the cytosolic tail of CD4 does not abrogate but rather enhances ER retention of CD4 upon expression of either wild-type Vpu or Vpu-S52,56N (Fig. 6). Since Vpu and cytosolic tail-less CD4 only overlap at their TMDs, direct or indirect interactions at this level must be the main determinant of ER retention. Indeed, a Vpu mutant containing a heterologous TMD failed to retain CD4 in the ER (Fig. 7), further supporting a role of TMD interactions in ER retention.

How could interaction with Vpu result in CD4 retention in the ER? We envisaged that assembly with Vpu could either mask an ER export signal on CD4, or confer on CD4 an intrinsic tendency of Vpu to reside in the ER. Our analysis ruled the first possibility unlikely, as CD4 has only a weak ER export signal in its cytosolic tail (Fig. 6). Moreover, even this weak signal can wrest a small amount of CD4 away from Vpu and out of the ER, as demonstrated by the higher ER retention of cytosolic tail-less CD4 relative to full-length CD4 in the presence of the Vpu-S52,56N mutant (when neither CD4 construct is degraded) (Fig. 6). Finally, replacement of the CD4 cytosolic tail by that of the VSV-G protein, which has a strong ER export signal [50], completely prevented ER retention of CD4 by Vpu (Fig. 8). Therefore, we favor the second explanation of retention *in trans*, according to which Vpu itself has ER retention information that is transmitted to CD4 upon assembly. Indeed, our immunofluorescence and immunoelectron microscopy analysis show that a large fraction of Vpu localizes to the ER (Figs. 3 and S3), irrespective of the presence or absence of CD4 (data not shown). CD4 may thus be detained in the ER by forming a transport-incompetent complex with Vpu.

The conveyance of ER retention information from Vpu to CD4, however, does not account for the full extent of CD4 retention in the ER. SCF^β-TrCP^-mediated ubiquitination on lysine and serine/threonine residues in the CD4 cytosolic tail provides an additional contribution to ER retention, as evidenced by the higher degree of retention elicited by NPL4 depletion (Fig. 3) relative to disruption of the Vpu-β-TrCP1/2 interaction (Fig. 4) or mutation of Ub acceptor residues in the CD4 cytosolic tail (Fig. 5). Recently, ubiquitination of a palmitoylation-deficient mutant of the lipoprotein receptor-related protein 6 (LRP6) was also shown to mediate ER retention of this protein [51]. The exact mechanism by which ubiquitination can impart ER retention of mutant LRP6 or CD4 in the presence of Vpu remains to be elucidated. Therefore, Vpu retains CD4 in the ER, independently of targeting to degradation, by the additive effects of two distinct mechanisms: TMD-mediated conferral of ER residency (accounting for ∼50% of the ER retention) and ubiquitination of the CD4 cytosolic tail (adding another ∼25% to ER retention).

### A Variant ERAD Pathway

In addition to retaining CD4 in the ER, Vpu targets newly-synthesized CD4 for proteasomal degradation [5], [16], [17]. This targeting has been proposed to be fundamentally distinct from ERAD, mainly based on a study using yeast as a heterologous expression system [23]. This study showed that the typical yeast ERAD components, Hrd1p, Hrd3p and Ubc7p, are dispensable for Vpu-induced CD4 degradation [23], a fact that our results have confirmed for the human orthologs, HRD1, SEL1L and UBC7, in human cells (Fig. 1). The participation of a non-ERAD Ub ligase, SCF^β-TrCP^, in Vpu-mediated CD4 degradation [21], [22] has further emphasized the distinctive nature of this process. However, we find that the VCP-UFD1L-NPL4 complex, a key component of the ERAD machinery, is involved in CD4 degradation by Vpu (Figs. 1 and 2). As previously shown for other ERAD substrates [37], [38], [39], [40], our results are consistent with VCP-UFD1L-NPL4 mediating extraction of CD4 from the ER membrane (Fig. 1). Vpu thus appears to bypass the early stages of ERAD, including substrate recognition and ubiquitination by ERAD machinery components, but joins in the later stages, beginning with dislocation by the VCP-UFD1L-NPL4 complex. We speculate that other components that act downstream of VCP-UFD1L-NPL4 in typical ERAD [28] might also participate in Vpu-induced CD4 degradation.

Over the past few years, it has become increasingly clear that there is not a single ERAD pathway but several alternative routes for targeting cellular proteins with defects in luminal (ERAD-L), membrane (ERAD-M), and cytosolic domains (ERAD-C) [28]. Similarly, various herpesviruses downregulate class I molecules of the major histocompatibility complex (MHC-I) from the ER by engaging the ERAD machinery at different levels. For example, the human cytomegalovirus (HCMV) immunoevasin, US11, establishes TMD interactions with Derlin-1 that are required for the ability of this viral protein to downregulate MHC-I [52]. In contrast, another HCMV immunoevasin, US2, downregulates MHC-I by a mechanism that is Derlin-1-independent [52] but involves cytosolic domain interactions with signal peptide peptidase [53]. The mK3 immunoevasin encoded by murine γ-herpesvirus 68 (MHV-68) exemplifies yet another variation on the mechanisms used by herpesviruses to downregulate MHC-I [54]. Unlike US11 and US2, mK3 has intrinsic Ub ligase activity that ubiquitinates newly-synthesized MHC-I, leading to its disposal by the proteasome [55]. Importantly, all of these mechanisms eventually merge at the level of the VCP-UFD1L-NPL4 complex for delivery to the proteasome. In this context, Vpu-induced CD4 downregulation represents another adaptation to the use of the same fundamental pathway for the proteasomal degradation of ER-retained proteins.

The activities of Vpu are not limited to the ER, but extend to other cellular compartments [10]. Indeed, Vpu has recently been shown to downregulate the restriction factor BST-2/tetherin from the cell surface [56], [57]. In light of these findings, it will be of interest to determine whether Vpu is capable of directly removing pre-existing CD4 from the cell surface in addition to targeting newly-synthesized CD4 to the ERAD pathway.

### Involvement of Cytosolic Tail Serine/Threonine Residues in CD4 Ubiquitination, ER Retention and ERAD Targeting

Mutation of all four lysines in the cytosolic tail of CD4 only partially inhibited Vpu-induced CD4 ubiquitination and degradation (Fig. 5). This led us to hypothesize that cytosolic tail residues other than lysine could be additional targets for ubiquitination. Previous studies showed that downregulation of MHC-I by mK3 involved ubiquitination of not only lysine, but also serine and threonine residues in the MHC-I cytosolic tail [55]. We tested whether this was also the case for Vpu-induced CD4 downregulation and found that more profound inhibition of CD4 ubiquitination and degradation could be achieved by mutation of all lysine, serine and threonine residues in the CD4 cytosolic tail (Fig. 5). Moreover, serine and threonine residues in the cytosolic tail of CD4 contributed to its retention in the ER mediated by Vpu (Fig. 5). These observations are highly significant because Vpu, unlike mK3, does not have intrinsic Ub ligase activity, indicating that serine/threonine residues could be ubiquitinated by a cellular enzyme. It will now be of interest to investigate whether this modification is mediated by SCF^β-TrCP^ or another Ub ligase, and whether other ERAD substrates undergo a similar modification.

## Materials and Methods

### Recombinant DNA Constructs

pcDNA3.1-FLAG-Ub was kindly provided by S. Ishikura (NICHD, NIH). pFLAG-CMV2-human β-TrCP1 was obtained from Y. Ben-Neriah (Hadassah Medical School, Hebrew University). pNLA-1 is a derivative of pNL4-3 [58], lacking the *gag* and *pol* genes but expressing all other viral genes. pCMV-human CD4 [20] and pcDNA3.1-codon-optimized Vpu (pcDNA3.1-Vphu) [41] were used as templates for site-directed mutagenesis using a QuikChange II kit (Stratagene, Cedar Creek, TX). pcDNA3.1 plasmids encoding RGS-His-tagged mouse wild-type VCP, VCP-ΔN, VCP-AA and VCP-QQ were previously reported [39], [59]. Mouse wild-type UFD1L, UFD1L-ΔUT3 (residues 215-307) and UFD1L-ΔUT6 (residues 1-214) cDNAs were amplified by PCR using pcDNA3.1-FLAG-mouse UFD1L [60] as template and cloned as XhoI/BamHI fragments into the pcDNA3.1/*myc*-His A vector (Invitrogen, Carlsbad, CA). To produce FLAG-tagged rat wild-type NPL4, NPL4-ΔUBD (residues 96-608) and NPL4-ΔZFD (residues 1-579), cDNAs were PCR-amplified from pFLAG-His-rat NPL4 [60] and cloned into the pFLAG-CMV-6c vector (Sigma-Aldrich, Saint Louis, MO) as EcoRI/BglII fragments. C-terminal FLAG-One-STrEP-tagged CD4 was cloned as an EcoRI/BamHI fragment into the pcDNA3.1/*myc*-His A vector. pCMV-human CD4 and pEGFP-N1-VSV-G [61] were used as templates in a sewing PCR strategy to generate the CD4-VSV-G-cyto chimeric cDNA (human CD4: bp 1–1260; VSV-G: bp 1447–1536), which was cloned as an EcoRI/NotI fragment into the pCI-Neo vector (Promega, Madison, WI). A sewing PCR approach was also used to create a cDNA encoding Vpu-VSV-G-TMD (Vpu: bp 1–12; VSV-G: bp 1393-1461; Vpu: 82–246), which was cloned into the pcDNA3.1/*myc*-His A vector as a EcoRI/XhoI fragment. All mutagenesis and cloning products were verified by DNA sequencing.

### Antibodies

The following mouse monoclonal antibodies were used in this study: 4B12 (Leica Microsystems, Bannockburn, IL), OKT4 (eBiosciences, San Diego, CA) or unconjugated and allophycocyanin (APC)-conjugated S3.5 (Caltag Laboratories, Burlingame, CA) to human CD4; Ab-5 to actin, clone 37 to calnexin and clone 19 to UFD1L (BD Biosciences, San Jose, CA); 58.13.3 to VCP (RDI Research Diagnostics, Concord, MA); H68.4 to human transferrin receptor (Zymed, San Francisco, CA); M2 to FLAG (Sigma-Aldrich). A mouse polyclonal antibody to NPL4 was obtained from Novus Biologicals (Littleton, CO). Rabbit polyclonal antibodies to FLAG and *myc* were from Sigma-Aldrich and Cell Signaling (Danvers, MA), respectively. Rabbit polyclonal antibodies to the human CD4 cytosolic tail (residues 420 to 447) [17] and Vpu (residues 32 to 81) [62] were previously described. Alexa Fluor 488-conjugated donkey anti-rabbit IgG (H+L) was from Molecular Probes (Eugene, OR). Alexa Fluor 594- or 647-conjugated donkey anti-mouse IgG2a or IgG1, respectively, were from Invitrogen. HRP-conjugated donkey anti-mouse IgG and donkey anti-rabbit IgG were from Amersham Biosciences (Piscataway, NJ).

### Cell Culture, Transfections and siRNA Treatment

HeLa cells (American Type Culture Collection, Manassas, VA) were transiently transfected by using Lipofectamine 2000 (Invitrogen). Plasmids encoding human CD4 (pCMV-CD4) and codon-optimized Vpu (pcDNA3.1-Vphu) were transfected at a 1∶1 ratio. Vpu levels driven from this codon-optimized construct were ∼3-fold higher than those driven from the proviral pNL4-3 construct (Fig. S4). However, similar levels of CD4 downregulation were attained with amounts of codon-optimized Vpu that were up to 16-fold lower (Fig. S4). ON-TARGET*plus* SMARTpool siRNAs and siControl duplex siRNA (Dharmacon, Lafayette, CO) at a final concentration of 100 nM were used to knockdown expression of endogenous ERAD targets (including β-TrCP1/2) and GAPDH, respectively (Table S1). Silencing was achieved by double transfection of 0.5×105 HeLa cells with Oligofectamine (Invitrogen). Cells were analyzed 48 h after the second round of transfection.

### Immunoprecipitation and Immunoblotting

Cells were lysed in ice-cold lysis buffer (0.5% Triton X-100, 50 mM Tris-HCl pH 7.5, 150 mM NaCl, 5 mM EDTA) supplemented with the complete Mini protease inhibitor cocktail (Roche Diagnostics, Indianapolis, IN). Equivalent amounts of cell lysates were subjected to immunoprecipitation as described [63]. Immunoprecipitated proteins or whole cell lysates (10 µg) were subjected to SDS-PAGE using the NuPAGE Bis-Tris Gel system (Invitrogen) and immunoblotting as previously described [64]. Membrane bound horseradish peroxidase (HRP)-conjugated antibodies were detected using the SuperSignal West Pico Chemiluminescent Substrate from Thermo Scientific (Rockford, IL). Data analysis was performed using the Image J software.

### Pulse-Chase Analysis

Cells grown in 6-well plates were incubated for 30 min at 37°C in methionine- and cysteine-free DMEM (Invitrogen), pulse-labeled with 0.2 mCi/ml [^35^S]methionine-cysteine (Express Protein Label; Perkin Elmer, Boston, MA) for 2 min at 37°C, and chased in complete medium (DMEM supplemented with 10% fetal bovine serum) supplemented with 5 mM L-methionine and L-cysteine (Sigma-Aldrich). Cellular ATP levels were depleted during the chase period using 20 mM 2-deoxy-D-glucose and 10 mM sodium azide (Sigma-Aldrich) in glucose-free DMEM (Invitrogen). At each chase time, cells were extracted by incubation in ice-cold lysis buffer (0.5% Triton X-100, 50 mM Tris-HCl pH 7.5, 300 mM NaCl, 5 mM EDTA) supplemented with a protease inhibitor cocktail, and lysates were then subjected to immunoprecipitation as described [63]. Immunoprecipitated proteins were analyzed by SDS-PAGE and visualized by fluorography on a Typhoon 9200 PhosphorImager (Amersham Biosciences). Data analysis and quantification was performed using the ImageQuant software.

### Subcellular Fractionation

Cell fractionation and membrane protein extraction with Na_2_CO_3_ were performed as previously described [65].

### Microscopy

Indirect immunofluorescence staining of fixed, permeabilized cells was performed as described [64]. Cells were examined with an Olympus FluoView FV1000 laser scanning confocal unit attached to an Olympus IX81 motorized inverted microscope as previously described [66]. Immunoelectron microscopy was performed as reported [14].

### *In Vivo* Ubiquitination

Immunoprecipitation of CD4 under denaturing conditions was performed as described [63]. Briefly, HeLa cells expressing FLAG- or *myc*-tagged Ub were lysed in a denaturing lysis buffer (1% SDS, 50 mM Tris-HCl pH 7.4, 5 mM EDTA, 10 mM dithiothreitol, 15 U/ml DNase I, 10 mM α-iodoacetamide, 5 mM N-ethylmaleimide) supplemented with the complete Mini protease inhibitor cocktail. After heating samples for 10 min at 100°C, the suspensions were diluted 10-fold in a non-denaturing lysis buffer (1% Triton X-100, 50 mM Tris-HCl pH 7.5, 150 mM NaCl, 5 mM EDTA, 10 mM α-iodoacetamide, 5 mM N-ethylmaleimide) supplemented with the complete Mini protease inhibitor cocktail. CD4 was immunoprecipitated using a conformation-independent anti-CD4 antibody. Alternatively, FLAG-One-STrEP-tagged CD4 was pulled-down using Strep-Tactin sepharose (IBA, Göttingen?, Germany). Ubiquitinated CD4 was then analyzed by immunoblotting with antibodies to the FLAG or *myc* epitopes, respectively.

### Endo H and PNGase F Digestion

Cell lysates made in 1% SDS, 0.1 M Tris-HCl pH 8 were processed for Endo H and PNGase F digestion as previously reported [67].

### Fluorescence-activated Cell Sorting (FACS)

Non-permeabilized cells were stained with an APC-conjugated antibody to the human CD4 ectodomain and prepared for FACS analysis as described [13].

### Accession Numbers

The HIV-1 Vpu and human CD4 clones used in this study correspond to Swiss-Prot entries P05923 and P01730, respectively.

## Supporting Information

Table S1 Cellular factors targeted in the siRNA screen.
(0.06 MB DOC)

Figure S1 CD4 half-life is dramatically shortened by Vpu in a VCP-dependent manner. (A, B) Determination of CD4 half-life. (A) HeLa cells expressing human CD4 were pulse-labeled for 2 min with [^35^S]methionine-cysteine and then chased for the indicated times at 37°C. Cell extracts were subjected to immunoprecipitation with an antibody to the CD4 cytosolic tail. (B) Percentage of CD4 at each chase time relative to CD4 at time 0 (100% control). The half-life of CD4 determined from this experiment was ∼4.8 h. (C, D) Phosphorylation of Vpu is essential for CD4 degradation. (C) HeLa cells were transfected with plasmids encoding human CD4 and no Vpu (empty-vector), wild-type Vpu or the non-phosphorylatable Vpu-S52,56N. At 12 h after transfection, cell extracts from cells treated as in A were subjected to immunoprecipitation with antibodies to the CD4 cytosolic tail and Vpu. (D) Percentage of CD4 at each chase time relative to CD4 at time 0 (100% control). (E, F) Analysis of CD4 stability in VCP-depleted cells expressing Vpu. (E) HeLa cells were treated without (mock) or with siRNAs to VCP. Cells were then transfected with plasmids encoding human CD4 and Vpu. At 12 h after transfection, cells were treated as in A followed by immunoprecipitation with an antibody to the CD4 cytosolic tail. (F) Percentage of CD4 at each chase time relative to CD4 at time 0 (100% control). In all experiments, immunoprecipitated species were analyzed by SDS-PAGE and fluorography.
(0.35 MB TIF)

Figure S2 CD4 ubiquitination depends on Vpu phosphoserines 52 and 56. (A) HeLa cells were transfected with plasmids encoding FLAG-One-STrEP-tagged human CD4 and *myc*-tagged Ub, plus no Vpu (empty vector), wild-type Vpu or Vpu-S52,56N (1∶0.5∶1 ratio of CD4, Ub and Vpu, respectively). At 12 h after transfection, equivalent amounts of cell lysates made under denaturing conditions were subjected to pull-down with Strep-Tactin-Sepharose. Ubiquitination of CD4 was detected by immunoblotting with a polyclonal antibody to the *myc* epitope. (B) CD4 levels in the presence of wild-type Vpu or Vpu-S52,56N from A were quantified by densitometry and expressed as percentage of the total amount of CD4 in the absence of Vpu (100% control). Data are represented as the mean ± SEM from three independent experiments. (C) Ub_n_-CD4 levels in the presence of wild-type Vpu or Vpu-S52,56N from A were quantified by densitometry and expressed as percentage of the total amount of Ub_n_-CD4 in the absence of Vpu (100% control). These values were normalized for the remaining CD4 in A (*i.e.*, 1 for Ub_n_-CD4 in the absence of Vpu). Data are the mean ± SEM from three independent experiments.
(0.39 MB TIF)

Figure S3 ER localization of Vpu and lack of effect on transferrin receptor stability, maturation and transport. (A-C) Specificity of Vpu localization to the ER. HeLa cells expressing Vpu were fixed and processed for immunoelectron microscopy. A Vpu-transfected and a Vpu-untransfected cell stained with an antibody to Vpu and a nanogold-conjugated secondary antibody were imaged in the same field of view. Notice the staining of the ER cisternae and the nuclear envelope in the transfected cell and the total absence of staining in the untransfected cell. N: nucleus; PM: plasma membrane. Bars: 1 µm. (D, E) Transferrin receptor (TfnR) stability, maturation and transport are not affected by Vpu expression. (D) Cell lysates from HeLa cells were digested with Endo H, PNGase F or left untreated before immunoblotting with an antibody to the TfnR cytosolic tail. (E) Data are represented as mean ± SEM from three independent experiments like that in D.
(0.63 MB TIF)

Figure S4 Expression of codon-optimized Vpu at levels comparable to those from proviral DNA are fully effective at downregulating CD4. (A) Codon-optimized Vpu is expressed at ∼3-fold higher levels than Vpu expressed from proviral DNA. HeLa cells were transfected with 2.5 µg of the proviral pNL4-3 (∼14.8 kb) or pNLA-1 (∼10.7 kb) plasmids, or with varying amounts of the pcDNA3.1-codon-optimized Vpu (pcDNA3.1-Vphu) (∼5.7 kb) construct, with 2.5 µg corresponding to a 1∶1 dilution. Total amounts of transfected DNA were kept constant in all samples by compensation with empty-vector DNA. At 12 h after transfection, cell extracts were subjected to immunoblotting with antibodies to Vpu and actin (used as a loading control). A 1∶1 dilution of pNL4-3 was found to yield Vpu expression levels equivalent to a 1∶8 dilution of pcDNA3.1-Vphu. Correcting for the different sizes of these plasmids (*i.e.*, comparing equivalent molar amounts), the Vpu expression level yielded by pcDNA3.1-Vphu was ∼3-fold higher than that from pNL4-3. (B–E) Similar decreases of both cell surface and total CD4 levels are attained with 1∶1 to 1∶16 dilutions of plasmid encoding Vphu. (B) HeLa cells were transfected with 2.5 µg of a construct encoding human CD4 and several dilutions of the pcDNA3.1-Vphu or pcDNA3.1-Vphu-S52,56N (*i.e.*, 2.5 µg = 1∶1 dilution). At 12 h after transfection, cell lysates were subjected to immunoblotting with antibodies to CD4, Vpu and actin (used as a loading control). Notice that increasing amounts of expressed Vphu-S52,56N did not affect CD4 expression and stability. (C) CD4 levels in the presence of Vphu were quantified by densitometry and expressed as percentage of the total amount of CD4 in the absence of Vphu (100% control). (D) HeLa cells transfected with 2.5 µg of a plasmid encoding human CD4 and different amounts of pcDNA3.1-Vphu (*i.e.*, 2.5 µg = 1∶1 dilution) were analyzed for cell surface CD4 by FACS. (E) Bar graphs represent percentage of surface CD4 levels in cells expressing Vphu relative to CD4-surface levels in the absence of Vphu (100%). Values are expressed as mean ± SEM from three independent experiments.
(1.05 MB TIF)
