# Supplementary material for: Multilayered Mechanism of CD4 Downregulation by HIV-1 Vpu Involving Distinct ER Retention and ERAD Targeting Steps
Source: PLoS Pathog. 2010 Apr 29;6(4):e1000869. doi: 10.1371/journal.ppat.1000869 (PMC2861688; doi:10.1371/journal.ppat.1000869)
Supplement: Table S1 — Cellular factors targeted in the siRNA screen. (0.06 MB DOC) [file ppat.1000869.s001.doc]

| ***Controls*** | | | |
| --- | --- | --- | --- |
| **Gene Symbol** | **Accession** | **Function** | **ON-TARGET*plus* siRNA** |
| GAPDH | NM_002046 | Metabolism | L-004253-00 |
| β-TrCP1 | NM_003939 | SCF^β-TrCP1^ E3 ubiquitin ligase | L-003463-00 |
| β-TrCP2 | NM_012300 | SCF^β-TrCP2^ E3 ubiquitin ligase | L-003490-00 |
|  | | | |
| ***ERAD Components*** | | | |
| **Gene Symbol** | **Accession** | **Function** | **ON-TARGET*plus* siRNA** |
| UBC6e | NM_016021 | E2 ubiquitin-conjugating enzyme | L-007266-00 |
| UBC7 | NM_003343 | E2 ubiquitin-conjugating enzyme | L-009095-00 |
| HRD1 | NM_032431 | HRD1-SEL1L E3 ubiquitin ligase | L-007090-00 |
| SEL1L | NM_005065 | HRD1-SEL1L E3 ubiquitin ligase | L-004885-00 |
| TEB4 | NM_005885 | E3 ubiquitin ligase | L-006925-00 |
| CHIP | NM_005861 | E3 ubiquitin ligase | L-007201-00 |
| GP78 | NM_001144 | GP78-RMA1 E3 ubiquitin ligase | L-006522-00 |
| Derlin-1 | NM_024295 | Retrotranslocation | L-010733-01 |
| Derlin-2 | NM_016041 | Retrotranslocation | L-010576-01 |
| Derlin-3 | NM_001002862 | Retrotranslocation | L-032237-01 |
| VIMP | NM_018445 | Retrotranslocation | L-019353-01 |
| UBXD2 | NM_014607 | Retrotranslocation | L-014184-01 |
| VCP | NM_007126 | Proteasomal targeting | L-008727-00 |
| UFD1L | NM_001035247 | Proteasomal targeting | L-017918-00 |
| NPL4 | NM_017921 | Proteasomal targeting | L-020796-01 |
| HHR23A | NM_005053 | Proteasomal targeting | L-005231-00 |

**Table S1. Cellular factors targeted in the siRNA screen.**
