# Supplementary figures and images for: Multilayered Mechanism of CD4 Downregulation by HIV-1 Vpu Involving Distinct ER Retention and ERAD Targeting Steps
Source: PLoS Pathog. 2010 Apr 29;6(4):e1000869. doi: 10.1371/journal.ppat.1000869 (PMC2861688; doi:10.1371/journal.ppat.1000869)

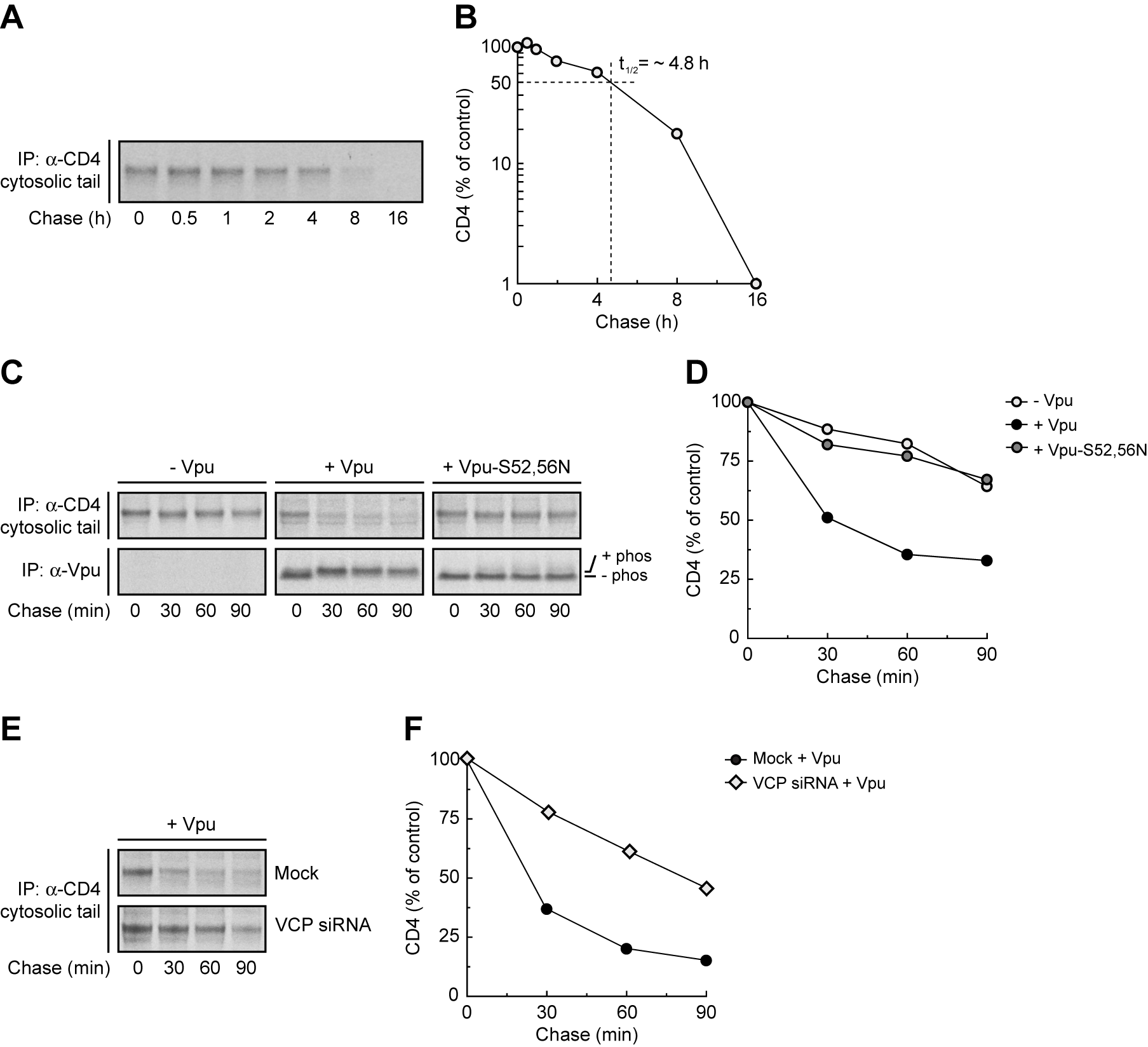

Supplement: Figure S1 — CD4 half-life is dramatically shortened by Vpu in a VCP-dependent manner. (A, B) Determination of CD4 half-life. (A) HeLa cells expressing human CD4 were pulse-labeled for 2 min with [35S]methionine-cysteine and then chased for the indicated times at 37°C. Cell extracts were subjected to immunoprecipitation with an antibody to the CD4 cytosolic tail. (B) Percentage of CD4 at each chase time relative to CD4 at time 0 (100% control). The half-life of CD4 determined from this experiment was ∼4.8 h. (C, D) Phosphorylation of Vpu is essential for CD4 degradation. (C) HeLa cells were transfected with plasmids encoding human CD4 and no Vpu (empty-vector), wild-type Vpu or the non-phosphorylatable Vpu-S52,56N. At 12 h after transfection, cell extracts from cells treated as in A were subjected to immunoprecipitation with antibodies to the CD4 cytosolic tail and Vpu. (D) Percentage of CD4 at each chase time relative to CD4 at time 0 (100% control). (E, F) Analysis of CD4 stability in VCP-depleted cells expressing Vpu. (E) HeLa cells were treated without (mock) or with siRNAs to VCP. Cells were then transfected with plasmids encoding human CD4 and Vpu. At 12 h after transfection, cells were treated as in A followed by immunoprecipitation with an antibody to the CD4 cytosolic tail. (F) Percentage of CD4 at each chase time relative to CD4 at time 0 (100% control). In all experiments, immunoprecipitated species were analyzed by SDS-PAGE and fluorography. (0.35 MB TIF) [file ppat.1000869.s002.tif]

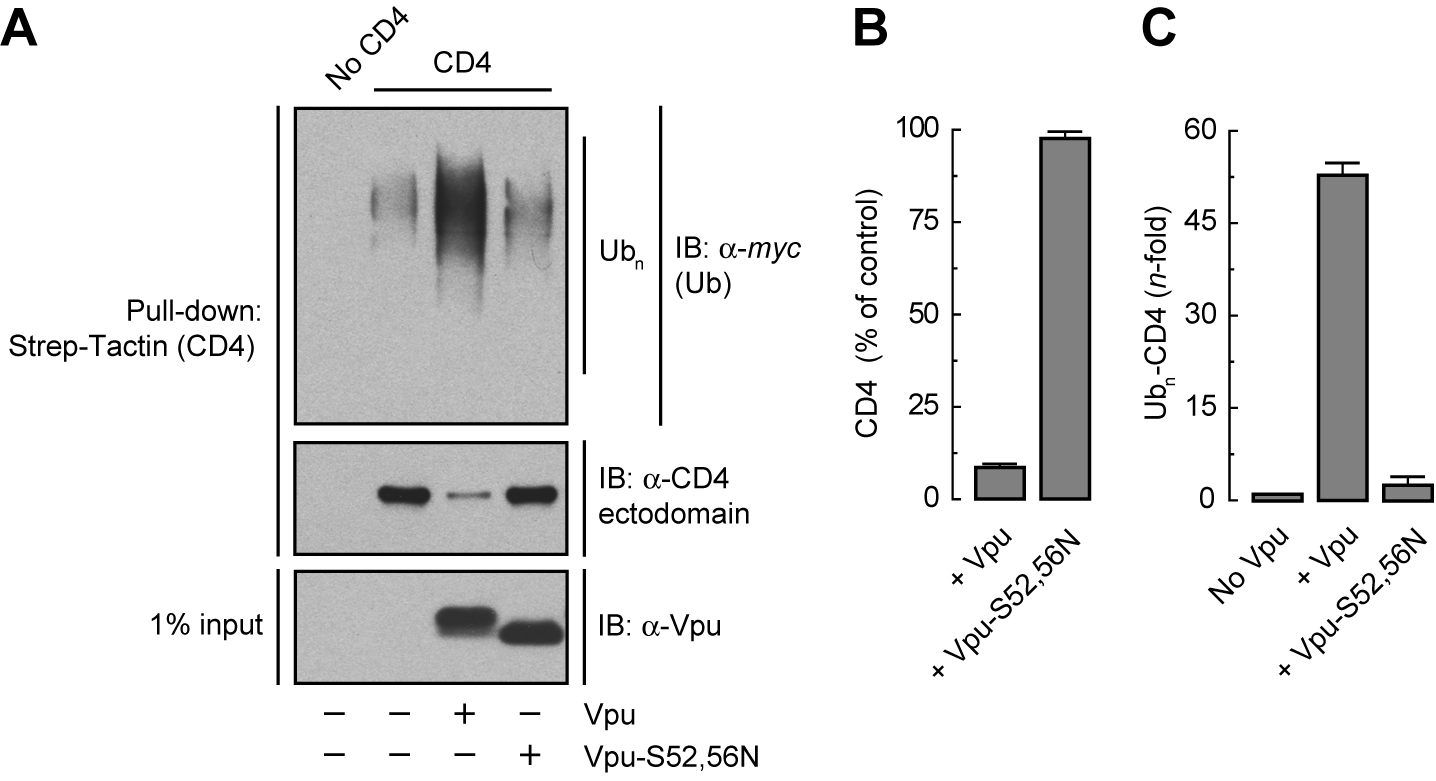

Supplement: Figure S2 — CD4 ubiquitination depends on Vpu phosphoserines 52 and 56. (A) HeLa cells were transfected with plasmids encoding FLAG-One-STrEP-tagged human CD4 and myc-tagged Ub, plus no Vpu (empty vector), wild-type Vpu or Vpu-S52,56N (1∶0.5∶1 ratio of CD4, Ub and Vpu, respectively). At 12 h after transfection, equivalent amounts of cell lysates made under denaturing conditions were subjected to pull-down with Strep-Tactin-Sepharose. Ubiquitination of CD4 was detected by immunoblotting with a polyclonal antibody to the myc epitope. (B) CD4 levels in the presence of wild-type Vpu or Vpu-S52,56N from A were quantified by densitometry and expressed as percentage of the total amount of CD4 in the absence of Vpu (100% control). Data are represented as the mean ± SEM from three independent experiments. (C) Ubn-CD4 levels in the presence of wild-type Vpu or Vpu-S52,56N from A were quantified by densitometry and expressed as percentage of the total amount of Ubn-CD4 in the absence of Vpu (100% control). These values were normalized for the remaining CD4 in A (i.e., 1 for Ubn-CD4 in the absence of Vpu). Data are the mean ± SEM from three independent experiments. (0.39 MB TIF) [file ppat.1000869.s003.tif]

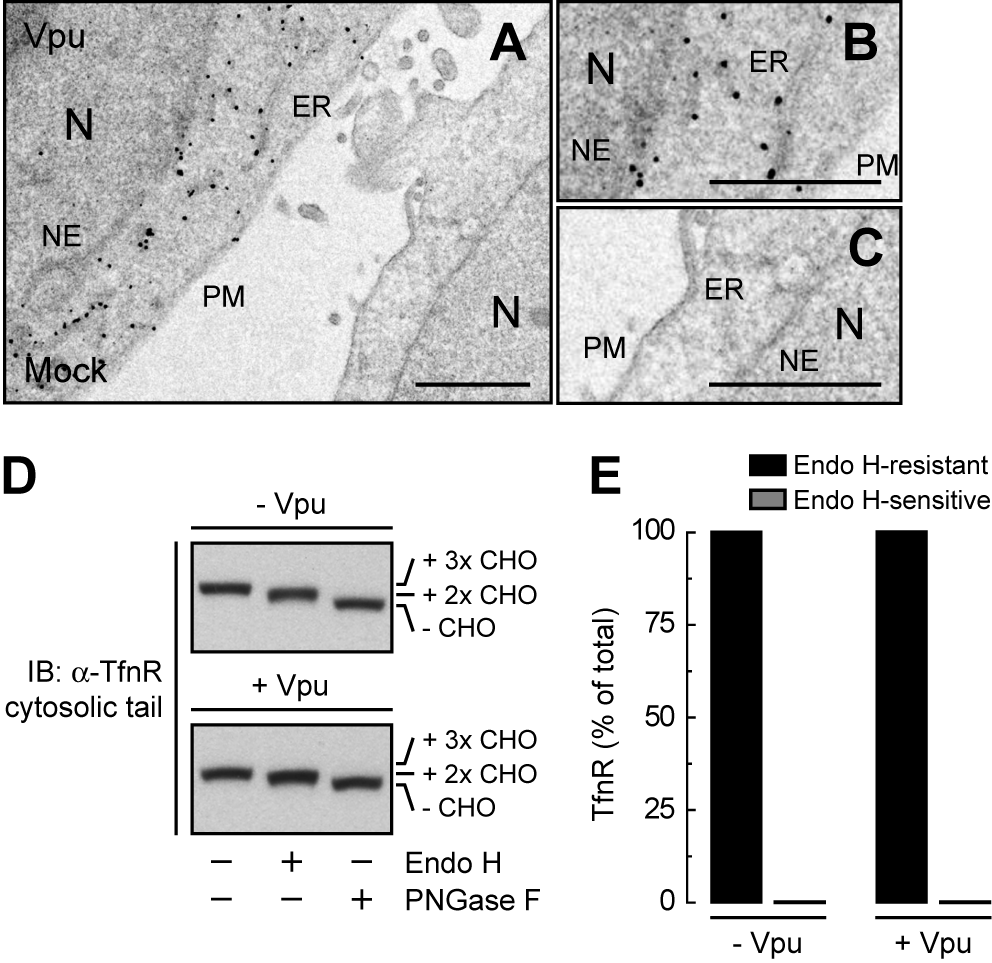

Supplement: Figure S3 — ER localization of Vpu and lack of effect on transferrin receptor stability, maturation and transport. (A-C) Specificity of Vpu localization to the ER. HeLa cells expressing Vpu were fixed and processed for immunoelectron microscopy. A Vpu-transfected and a Vpu-untransfected cell stained with an antibody to Vpu and a nanogold-conjugated secondary antibody were imaged in the same field of view. Notice the staining of the ER cisternae and the nuclear envelope in the transfected cell and the total absence of staining in the untransfected cell. N: nucleus; PM: plasma membrane. Bars: 1 µm. (D, E) Transferrin receptor (TfnR) stability, maturation and transport are not affected by Vpu expression. (D) Cell lysates from HeLa cells were digested with Endo H, PNGase F or left untreated before immunoblotting with an antibody to the TfnR cytosolic tail. (E) Data are represented as mean ± SEM from three independent experiments like that in D. (0.63 MB TIF) [file ppat.1000869.s004.tif]

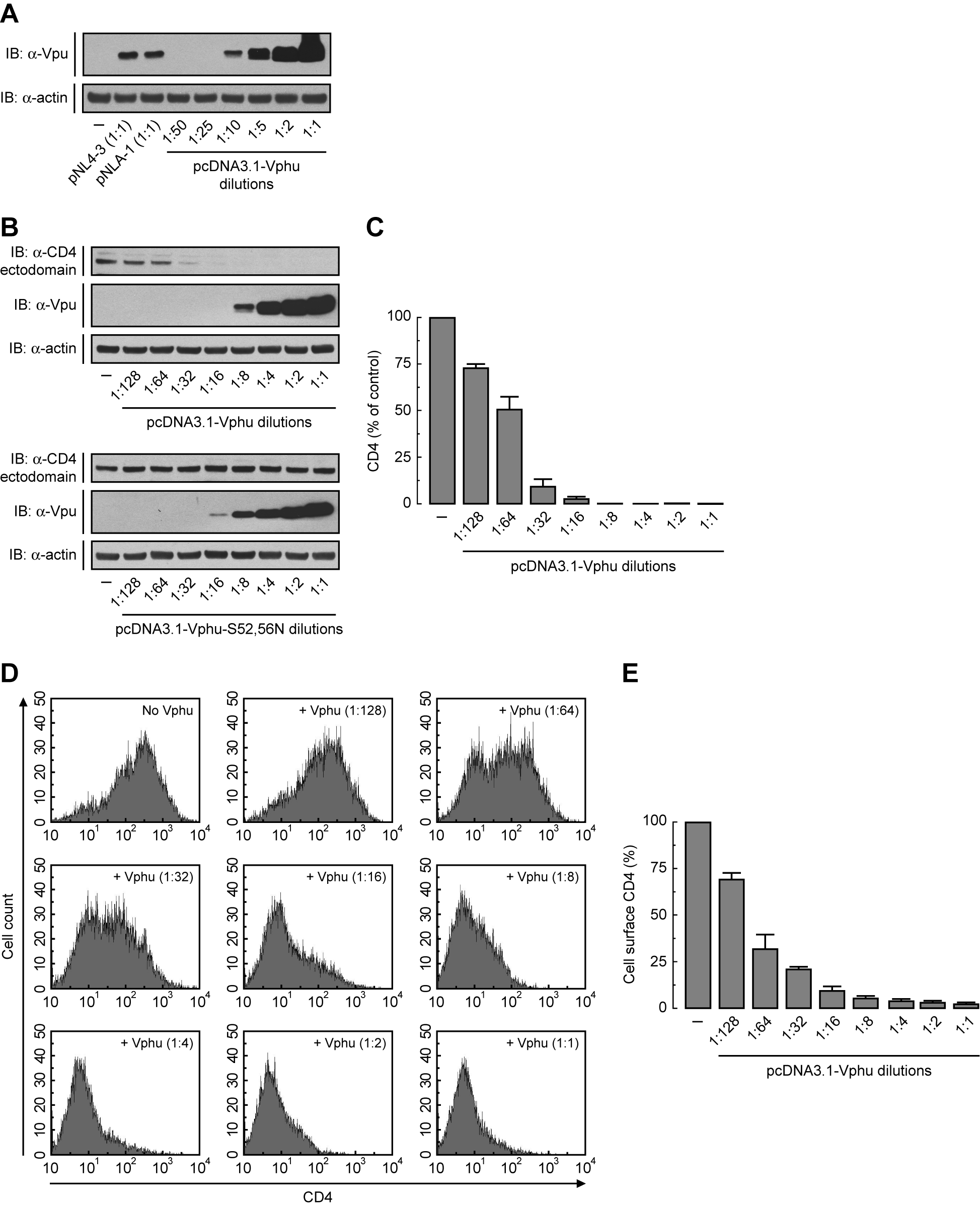

Supplement: Figure S4 — Expression of codon-optimized Vpu at levels comparable to those from proviral DNA are fully effective at downregulating CD4. (A) Codon-optimized Vpu is expressed at ∼3-fold higher levels than Vpu expressed from proviral DNA. HeLa cells were transfected with 2.5 µg of the proviral pNL4-3 (∼14.8 kb) or pNLA-1 (∼10.7 kb) plasmids, or with varying amounts of the pcDNA3.1-codon-optimized Vpu (pcDNA3.1-Vphu) (∼5.7 kb) construct, with 2.5 µg corresponding to a 1∶1 dilution. Total amounts of transfected DNA were kept constant in all samples by compensation with empty-vector DNA. At 12 h after transfection, cell extracts were subjected to immunoblotting with antibodies to Vpu and actin (used as a loading control). A 1∶1 dilution of pNL4-3 was found to yield Vpu expression levels equivalent to a 1∶8 dilution of pcDNA3.1-Vphu. Correcting for the different sizes of these plasmids (i.e., comparing equivalent molar amounts), the Vpu expression level yielded by pcDNA3.1-Vphu was ∼3-fold higher than that from pNL4-3. (B–E) Similar decreases of both cell surface and total CD4 levels are attained with 1∶1 to 1∶16 dilutions of plasmid encoding Vphu. (B) HeLa cells were transfected with 2.5 µg of a construct encoding human CD4 and several dilutions of the pcDNA3.1-Vphu or pcDNA3.1-Vphu-S52,56N (i.e., 2.5 µg = 1∶1 dilution). At 12 h after transfection, cell lysates were subjected to immunoblotting with antibodies to CD4, Vpu and actin (used as a loading control). Notice that increasing amounts of expressed Vphu-S52,56N did not affect CD4 expression and stability. (C) CD4 levels in the presence of Vphu were quantified by densitometry and expressed as percentage of the total amount of CD4 in the absence of Vphu (100% control). (D) HeLa cells transfected with 2.5 µg of a plasmid encoding human CD4 and different amounts of pcDNA3.1-Vphu (i.e., 2.5 µg = 1∶1 dilution) were analyzed for cell surface CD4 by FACS. (E) Bar graphs represent percentage of surface CD4 levels in cells expressing Vphu [file ppat.1000869.s005.tif]
